# Highly selective oxygen reduction to hydrogen peroxide on transition metal single atom coordination

**DOI:** 10.1038/s41467-019-11992-2

**Published:** 2019-09-05

**Authors:** Kun Jiang, Seoin Back, Austin J. Akey, Chuan Xia, Yongfeng Hu, Wentao Liang, Diane Schaak, Eli Stavitski, Jens K. Nørskov, Samira Siahrostami, Haotian Wang

**Affiliations:** 1 Rowland Institute, Harvard University, Cambridge, MA 02142 USA; 2 SUNCAT Center for Interface Science and Catalysis, Department of Chemical Engineering, Stanford University, Stanford, CA 94305 USA; 3 Center for Nanoscale Systems, Harvard University, Cambridge, MA USA; 4 Canadian Light Source Inc., University of Saskatchewan, Saskatoon, SK S7N 2V3 Canada; 5 Kostas Research Institute, Northeastern University, Burlington, MA 01803 USA; 6 National Synchrotron Light Source II, Brookhaven National Laboratory, Upton, NY 11973 USA; 7 SUNCAT Center for Interface Science and Catalysis, SLAC National Accelerator Laboratory, 2575 Sand Hill Road, Menlo Park, CA 94025 USA; 8 Department of Chemistry, University of Calgary, 2500 University Drive NW, Calgary, Alberta T2N 1N4 Canada; 9 Department of Chemical and Biomolecular Engineering, Rice University, Houston, TX 77005 USA

**Keywords:** Electrocatalysis, Sustainability, Materials science

## Abstract

Shifting electrochemical oxygen reduction towards 2e^–^ pathway to hydrogen peroxide (H_2_O_2_), instead of the traditional 4e^–^ to water, becomes increasingly important as a green method for H_2_O_2_ generation. Here, through a flexible control of oxygen reduction pathways on different transition metal single atom coordination in carbon nanotube, we discovered Fe-C-O as an efficient H_2_O_2_ catalyst, with an unprecedented onset of 0.822 V versus reversible hydrogen electrode in 0.1 M KOH to deliver 0.1 mA cm^−2^ H_2_O_2_ current, and a high H_2_O_2_ selectivity of above 95% in both alkaline and neutral pH. A wide range tuning of 2e^–^/4e^–^ ORR pathways was achieved via different metal centers or neighboring metalloid coordination. Density functional theory calculations indicate that the Fe-C-O motifs, in a sharp contrast to the well-known Fe-C-N for 4e^–^, are responsible for the H_2_O_2_ pathway. This iron single atom catalyst demonstrated an effective water disinfection as a representative application.

## Abstract (web summary)

Hydrogen peroxide can be produced alternatively using green inputs such as air, water, and sunlight but this requires a selective catalyst. Here the authors report an iron single atom catalyst that can convert oxygen into hydrogen peroxide with a selectivity of above 95% in both alkaline and neutral pH.

## Introduction

Molecular oxygen (O_2_) can be electrochemically reduced to water (H_2_O) via a 4e^–^ pathway, or hydrogen peroxide (H_2_O_2_) with 2e^–^ transferred in aqueous solutions. The former pathway is preferred in fuel cell applications to maximize the energy conversion efficiencies^1–3^, and the latter one represents a green synthetic method for H_2_O_2_^4–7^. Compared to the traditional energy, capital, and waste intensive anthraquinone process^6,8,9^, electrochemical synthesis of H_2_O_2_ becomes a promising alternate with significant advantages including: (1) mild reaction conditions under room temperature and ambient pressure; (2) renewable electricity as the energy source without fossil fuel consumptions; and (3) green precursors starting with water and air. Although a variety of highly active catalysts driving the 4e^–^ oxygen reduction reaction (ORR) have been developed to improve the performance of fuel cells^10–14^, there are less known catalysts that can selectively reduce O_2_ to H_2_O_2_ including noble metals^5,15–19^ and carbon materials^20–29^. A molecular level understanding of the elementary reaction steps could provide important guidance to the design of ORR catalysts for different pathways. The critical knob for searching efficient ORR catalysts relies on the proper binding strength between the reaction site and O species^27–29^. Taking the selective ORR to H_2_O_2_, where the O–O bond in O_2_ needs to be preserved, as an example here, a too-strong interaction between the reaction site and O species could easily dissociate the O_2_ molecule and direct the selectivity towards H_2_O, while a too-weak one may create a high reaction barrier to overcome^4,27^. Therefore, a materials platform with flexible tunability in electronic structures is highly desired for systematic control of ORR pathways as well as improvements in catalytic activities.

Transition-metal (TM) single atom motifs coordinated in well-defined carbon matrix, with a variety of tuning knobs such as the different metal atom centers and the varied adjacent coordinative dopants, have been attracting considerable interests in heterogeneous catalysis field^30–33^. This is mainly due to their unique electronic properties compared to their bulk metal counterparts for extraordinary activities, as well as their capability in tuning the binding strength with reaction intermediates for boosting desired catalytic pathways^34–36^. One representative example is the Ni single atom catalyst in our previous CO_2_ reduction studies where the binding with CO was significantly weakened on isolated Ni atomic sites compared to that of bulk Ni surface to facilitate CO evolution^37–39^. With potentially a wide range of tunability in binding with O species via different TM single atom coordination, this class of materials as a powerful platform thus provides us with great opportunities in exploring highly selective and active catalysts for H_2_O_2_ generation, as well as a flexible control of ORR pathways.

Here we report the TM single atom coordination motifs for a full range control of ORR pathways from the 2e^–^ reduction selectively to H_2_O_2_ towards the 4e^–^ to H_2_O. A series of TM single atoms including Fe, Pd, Co, and Mn are anchored into carbon nanotube (TM-CNT) vacancies (Fe-CNT, Pd-CNT, Co-CNT, and Mn-CNT, respectively) with neighboring C, O, or N coordination for pathway tuning. Among the different TMs, Fe-CNT presents the state-of-the-art performance towards H_2_O_2_ generation in terms of activity and selectivity. An unprecedented onset potential to reach 0.1 mA cm^−2^ H_2_O_2_ generation current is achieved at only 0.822 V versus reversible hydrogen electrode (vs. RHE) in 0.1 M KOH on rotating ring-disc electrode (RRDE), while a maximum H_2_O_2_ selectivity of more than 95% is delivered in both alkaline and neutral pH. With the O_2_ mass transport facilitated by a gas diffusion layer (GDL) electrode, the H_2_O_2_ generation rate by Fe-CNT can reach to 43 mA cm^−2^ with a 95.4% selectivity under only 0.76 V. By switching the neighboring O with N coordination, the 2e^–^ ORR pathway is successfully shifted towards 4e^–^ of H_2_O, demonstrating a wide range of reaction tunability in this materials platform. Density functional theory (DFT) calculations suggest that the catalytically active C and Fe sites in Fe–C–O and Fe–C–N motifs are responsible for the H_2_O_2_ and H_2_O pathways, respectively. In a variety of Fe–C–O motifs calculated, the incorporation of Fe atoms significantly improves their catalytic activities for H_2_O_2_ generation compared to those with only O dopants. As a prototype demonstration of potential applications, this high-performance H_2_O_2_ generation catalyst enables an effective water disinfection of >99.9999% bacteria removal at a treating rate of 125 L h^−1^ m^−2^_electrode_.

## Results

### Synthesis and characterizations of single atom catalysts

**Fig. 1:**
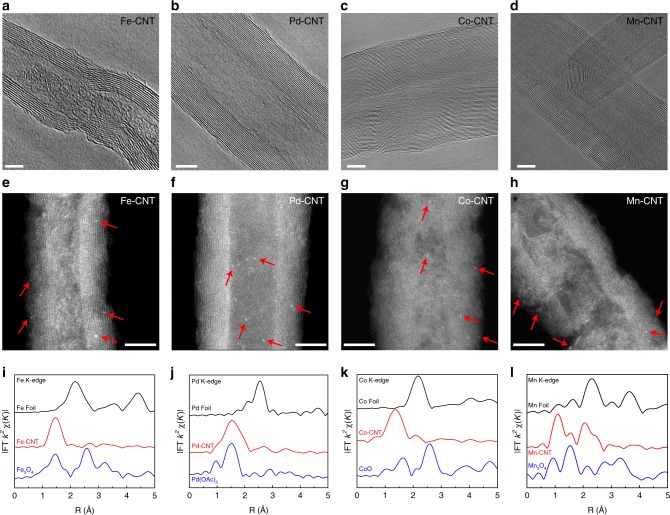
TEM and XAS characterizations of M-CNT catalysts. High resolution TEM and aberration-corrected HAADF-STEM images of **a**, **e** Fe-, **b**, **f** Pd-, **c**, **g** Co-, **d**, **h** Mn-CNT are shown in upper and middle panels, respectively, in which multiple rolled graphene layers can be clearly distinguished with no metal clusters/particles observed in each sample. The bright dots in HAADF-STEM (marked by red arrows) represent some typical metal single atoms. Scale bars, 5 nm. **i**–**l** Corresponding Fourier transformed EXAFS spectra of M-CNTs are plotted in R space at the bottom panel in comparison to their bulk metal foil and metal oxide control samples

A small quantity of TM cations (~0.1 at%) were first dispersed onto commercial surface-functionalized CNTs as the carbon matrix suspended in water (Supplementary Fig. 1), followed by freeze-dry and thermo annealing under Ar flow at 600 °C (Methods, Supplementary Note 1 and Supplementary Figs. 2–3)^38^. All four types of TM-CNT samples, including Fe, Pd, Co, and Mn, have shown similar structures in Fig. 1 by transmission electron microscopy (TEM) and aberration-corrected high-angle annular dark-field scanning TEM (HAADF-STEM). No nanoparticles or clusters were observed in the bright field TEM images by different scales (Fig. 1a–d; Supplementary Figs. 4–7), suggesting a well dispersion of TM atoms. Isolated TM atoms can be resolved by HAADF-STEM due to their high Z-contrast compared to those neighboring light elements such as C or O^32^. While all four isolated metal atoms were observed as the white dots in Fig. 1e–h, Pd-CNT presents the most distinguishable single atoms due to its heaviest atomic mass compared to the other three metal elements.

Although no obvious TM signals were detected by X-ray photoelectron spectroscopy (XPS, Supplementary Fig. 8) due to the low mass loading, synchrotron-based X-ray absorption spectroscopy (XAS) on the K-edge of Fe^40^, Pd^41^, Co^42^, and Mn^43^ can instead provide direct evidence of the valence states and coordination environments^38^. The X-ray absorption near-edge structure (XANES) spectra in Supplementary Fig. 9 indicate the partial-oxidation states of these metal dopants where the pre-edge peaks are located between the corresponding metal foils and metal oxides. This is mainly due to the strong M–C and M–O bonds via which the electrons from the metal atomic centers are partially depleted to the neighboring C and O sites, in a good agreement with the simulated charge density distributions and Balder charge analysis (Supplementary Table 1)^44^. In addition, the oxidation state of coordinated Fe is lower than simply adsorbed Fe on CNT, suggesting the different chemical environment between the adsorption case and coordination case (Supplementary Note 2, Supplementary Fig. 10). The corresponding Fourier transforms of extended X-ray absorption fine structure (EXAFS) for each metal are plotted in Fig. 1i–l. It is noted that the signals from bulk metal foils are dominated by M-M bonds, i.e., 2.17 Å for Fe–Fe, 2.55 Å for Pd–Pd, 2.18 Å for Co–Co and 2.31 Å for Mn–Mn, respectively^40–43^. In contrast, M–C or M–O bonds at ~1.4–2.0 Å are the major features in M-CNTs, reinforcing the isolated distribution of these TM single atoms in the carbon matrix. A shoulder peak at 2.05 Å is observed for Mn-CNT, which is corresponding to the Mn–O coordination^43^.

**Fig. 2:**
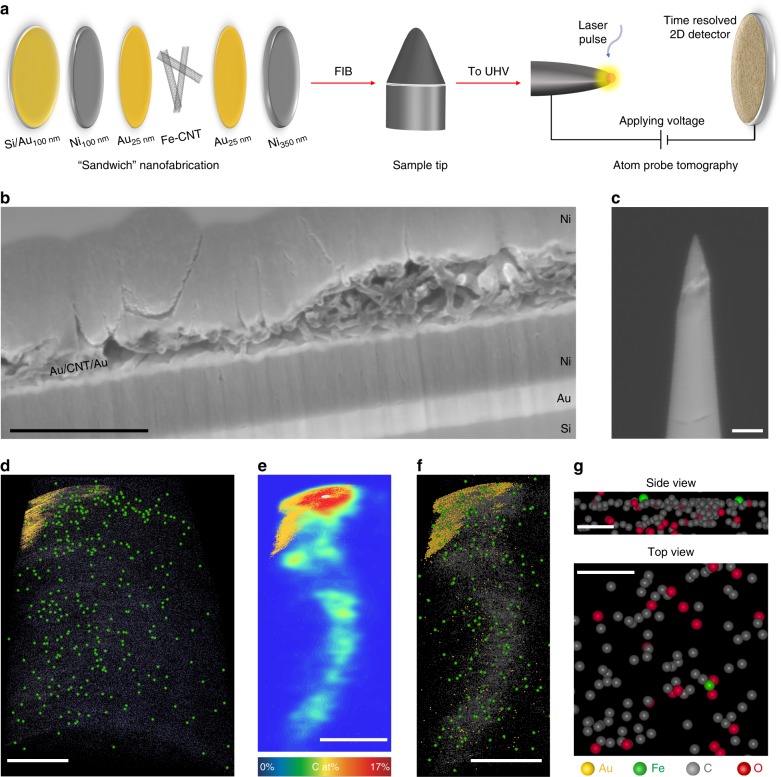
Atom probe tomography of Fe-CNT catalyst. **a** The schematic of the nanofabrication procedure of APT sample tips. **b** The cross-section SEM image of a catalyst sandwich after FIB cutting. **c** SEM image of the final APT specimen tip anchored on a Si microtip after sharpening. **d** Reconstructed APT data for a region containing Fe-CNT, with (**e**) a 2D contour plot of C atomic concentration and **f** the corresponding reconstruction for a region of 40 × 40 × 80 nm surrounding a CNT. **g** Side and top view of CNT planes, where single Fe atoms may have direct coordination with both C and O. The vacant space is mainly due to the low detection efficiency of atoms. Atom labels, Au in yellow, Fe in green, C in gray, and O in red in **d**–**g**; Scale bars, 500 nm in **b**, 200 nm in **c**, 20 nm in **d**–**f**, and 2 nm in **g**

Due to the capabilities in both three dimensional (3D) imaging and chemical composition measurements at the atomic scale, atom probe tomography (APT) becomes a complementary characterization to STEM and XAS to reveal additional information about the atomic structure in catalysts^37,45–48^. Fe-CNT was analyzed as a representative of other M-CNTs. We used a “sandwich” approach for the sample tip preparation (Fig. 2). We drop-casted dispersed Fe-CNT onto a nickel-coated substrate, sandwiched in between two 25-nm gold layers deposited by sputter-coating; the gold serves as a marker for the original surface of the CNT powder (Fig. 2a, Supplementary Fig. 11). The Au-CNT-Au sandwich is itself enclosed in layers of sputter-deposited Ni, to give sufficient material supports for APT tip preparation. Focused ion beam (FIB) procedures are applied to cut the sample into pieces, with a corresponding cross-section structure shown in the SEM image of Fig. 2b. Preparation of an APT-compatible needle-shaped specimen is carried out by FIB as shown in Fig. 2c. APT was then performed on a Cameca LEAP 4000 HR as detailed in the Methods, with a total of 9 million atoms collected. The reconstructed tomography of the sample tip is presented in Supplementary Movie 1, with the projected 2D image shown in Fig. 2d. Each dot represents the position of the ion detected by mass spectrometry, with a maximum detection efficiency of 38% (reflectron atom probe). Au layer as a marker in yellow is observed in the upper left corner, indicating the catalyst region for analysis. A few hundreds of isolated ^56^Fe atoms (identified only from the 56 Da peak due to molecular fragment overlaps at the other Fe isotopes and charge states) were well dispersed across the sample tip and highlighted in green. A smaller region attached to the Au marker was selected for detailed analysis as shown by the 2D contour map of carbon atom distribution (Fig. 2e), where the shape of a CNT was resolved. Fe atoms were distributed uniformly along the CNT as shown in the corresponding 2D projection (Fig. 2f), with no neighboring Fe atoms observed with a distance less than 3 Å. This is in consistent with STEM and XAS characterizations and further confirms the atomic dispersion of Fe in CNT. While a closer observation around Fe atoms is not able to provide the exact atomic structures due to the loss of ion signals during the detection process as well as the APT resolution (0.1–0.3 nm), it could still give us important hints of the possible coordination environment: the side view suggests a few atomic layers where Fe single atoms were anchored; the top view of one atomic layer, although with vacant space since a portion of atoms were not detected, shows that the isolated Fe atoms may have neighboring coordination with both C and O, suggesting a possible coordination of Fe–C–O motifs which will be further discussed in our theoretical calculations.

### Electrocatalytic ORR characterizations

**Fig. 3:**
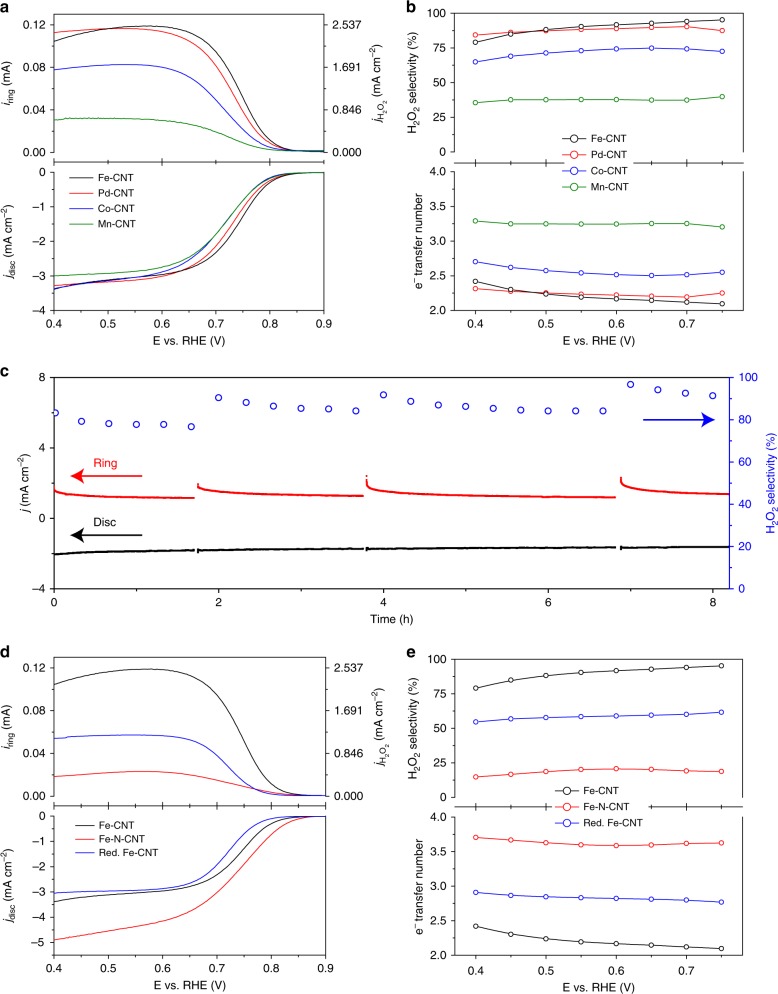
ORR performance of M-CNT catalysts cast RRDE in 0.1 M KOH. **a** Linear sweep voltammetry (LSV) of CNT background and Fe-, Pd-, Co-, Mn-CNT catalysts recorded at 1600 rpm and a scan rate of 5 mV s^−1^, together with the detected H_2_O_2_ currents on the ring electrode (upper panel) at a fixed potential of 1.2 V vs. RHE. **b** Calculated H_2_O_2_ selectivity and electron transfer number during potential sweep. **c** Stability measurement of Fe-CNT at a fixed disk potential of ~0.71 V. The ring electrode was refreshed several times by rapid scan at negative potentials to remove the accumulated PtO_x_ in continuous operation. **d**, **e** LSV and corresponding H_2_O_2_ selectivity comparison on Fe-CNT, Fe-N-CNT, and forming gas reduced Fe-CNT (Red. Fe-CNT) catalysts

The ORR performances were evaluated in 0.1 M KOH by casting a thin catalyst layer onto rotation ring disk electrode (RRDE), with the collection efficiency pre-calibrated by the redox reaction of [Fe(CN_6_)]_4_^−^/[Fe(CN_6_)]_3_^−^ (Supplementary Fig. 12, Methods). The potential of reference electrode was double confirmed by purging pure H_2_ gas onto a physically and electrochemically polished polycrystalline Pt wire or Pt rotation disc electrode at a reasonable rotation speed (Supplementary Fig. 13, Methods). The ORR peak of Fe-CNT was observed in the cyclic voltammetry in O_2_-saturated electrolyte, in contrast with the double layer current when O_2_ was switched to N_2_ (Supplementary Fig. 14). Figure 3a shows the polarization curves of M-CNTs for their performance screening at a constant catalyst loading of 0.1 mg cm^−2^, together with the H_2_O_2_ generation current detected by the Pt ring electrode (Methods). Note that the possible H_2_O_2_ decomposition on metal oxides compared to the generation should be negligible (Supplementary Note 3). The corresponding H_2_O_2_ selectivity and electron transfer numbers were plotted in Fig. 3b as a function of potential. The background performance of CNT support (O content of 4.5%) showed similar H_2_O_2_ selectivity compared to previous studies (Fig. 3a, b)^27^, which was significantly improved or decreased with TM single atoms incorporated. Note that the O contents in CNT were barely changed after the doping of metal atoms (Supplementary Fig. 8), ruling out its contribution to the changes of H_2_O_2_ selectivity. In addition, we excluded the possibility that the ratio between C and O might be influenced by adsorbed carbon dioxide and oxygen gas from air (Supplementary Fig. 15). Among those different TMs, Fe-CNT presents the best H_2_O_2_ generation performance evaluated by RRDE, with a maximal H_2_O_2_ selectivity of more than 95%, and a high potential of 0.822 V *vs*. RHE to deliver a 0.1 mA cm^−2^ H_2_O_2_ onset current. This early onset is superior to the so-far reported H_2_O_2_ catalysts as listed in the performance chart (Supplementary Fig. 16)^5,18,22–25^, such as Pd-Hg (0.72 V)^15^, Au-Pd (0.54 V)^17^, Pt single atoms (0.71 V)^16^, and highly oxidized CNTs (0.75 V)^27^, representing a facile ORR kinetics with negligible overpotential for O_2_-to-H_2_O_2_ conversion^5,19,23–26]^. By switching the metal dopants from Fe to Pd, Co, and Mn, the H_2_O_2_ selectivity was changed to 90.3, 74.8, and 39.8%, respectively, suggesting a wide range tuning of electron transfer numbers from 2.09 to 3.20. Supplementary Figure 17 shows the effects of Fe atom loading (0, 0.05, 0.1, and 0.2 at%) on H_2_O_2_ activity and selectivity. Compared to bare CNT, the performance was gradually increased with the increase of Fe atom loading, but dramatically dropped once Fe clusters was formed (Supplementary Note 1 and Supplementary Fig. 2), demonstrating the critical role of atomically dispersed Fe. Fe-CNT maintains its high H_2_O_2_ selectivity and activity when applied onto a GDL electrode with facilitated O_2_ mass diffusion for large current densities in electrolyzer, where the colorimetric quantification of H_2_O_2_ was employed instead (Supplementary Fig. 18, Methods). In 1 M KOH, the catalyst delivered a steady-state H_2_O_2_ partial current of 43 mA cm^−2^ at 0.76 V with a Faradaic efficiency of 95.4%, corresponding to a H_2_O_2_ production rate of ~1.6 mol g^−1^ h^−1^ or 8 mol m^−2^ h^−1^ (Supplementary Fig. 19). The catalytic activity of Fe-CNT in both RRDE test and bulk electrolysis presents significant improvements compared to previous studies of oxidized carbon catalysts (Supplementary Fig. 20). The performance stability of Fe-CNT single atom catalyst was also demonstrated on RRDE in Fig. 3c, with a stable H_2_O_2_ selectivity of above 90% over the 8-h continuous operation (Methods). Post-catalysis XAS analysis of Fe K-edge XANES overlaps well with that of pristine Fe-CNT, suggesting that the electronic structure and coordination of Fe single atoms remains unchanged (Supplementary Fig. 21). The corresponding Fourier transformed EXAFS spectrum of post-catalysis Fe-CNT reveals that Fe atoms still maintain an atomic dispersion. The reaction pathway can also be tuned by maintaining the metal center while switching its neighboring metalloid coordination, which combined with the corresponding changes in catalytic performances could further reveal the possible active coordination motifs for H_2_O_2_ generation. The H_2_O_2_ selectivity of Fe-CNT was significantly decreased to a maximum of 60% when the catalyst was annealed in forming gas with Fe–C–O coordination reduced (Red. Fe-CNT); the 4e^–^ ORR pathway was preferred when O was replaced with N to form Fe–C–N coordination, with the electron transfer number boosted from 2.09 of Fe-CNT to 3.71 of Fe-N-CNT^13,49–52^, and even to 3.90 when the mass loading was increased to a typical fuel cell test condition (Supplementary Fig. 22)^13,53^.

### Theoretical calculations

**Fig. 4:**
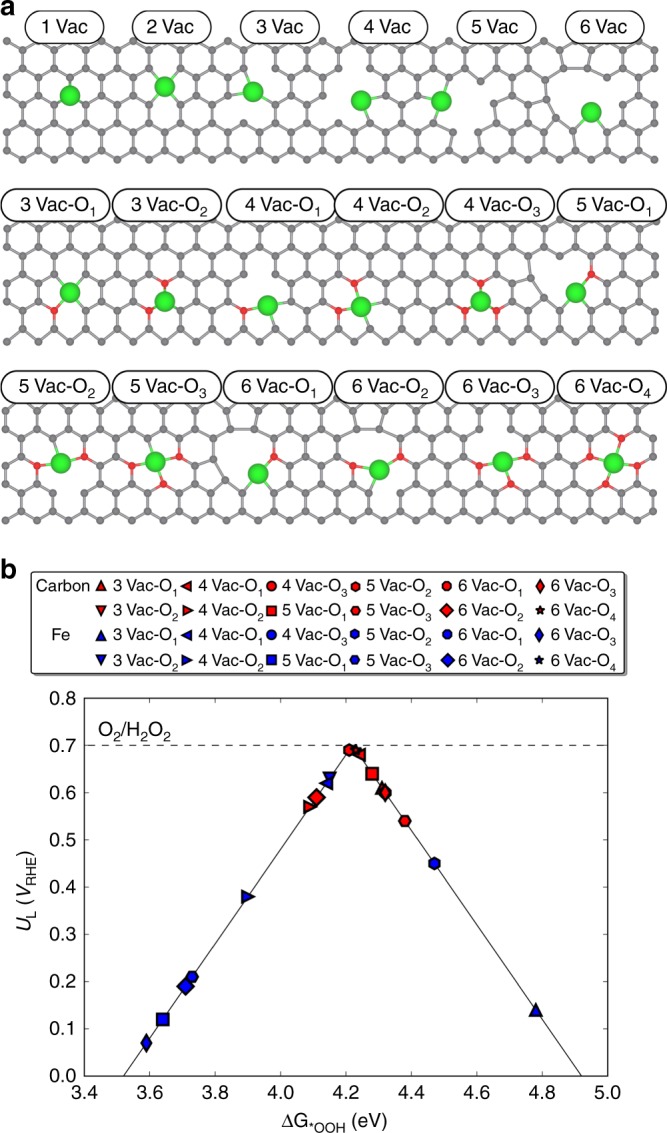
DFT calculations of the ORR activity and selectivity on different motifs. **a** All examined configurations for single Fe atom coordinated in two-dimensional carbon material with and without O species. We used the most stable surface coverage as a reference (Supplementary Figs. 29–31). Green, red, and gray colors denote Fe, O (or N), and C atoms, respectively. **b** The calculated ORR activity volcano plot for 2e^–^ pathway to H_2_O_2_. Red and blue symbols indicate *OOH adsorption at C and Fe, respectively. The equilibrium potential of O_2_/H_2_O_2_ is shown as a black dashed line. Some of the points are not shown in the volcano plot since their binding energies are out of the range

The above experiments suggested that the product selectivity of ORR could be related with the coordination motifs of Fe–C–O or Fe–C–N in those single atom catalysts. While based on current experimental techniques it is still challenging to directly resolve all of the exact atomic structures, density functional theory (DFT) calculations of different configurations (a total of 18 configurations as shown in Fig. 4a) become of great importance to understand the origin of selectivity change, and for rational design of new catalysts in the future. We used a two-dimensional graphene to model the CNT support used here, since the difference in binding free energies of reaction intermediates at CNT with a diameter larger than 20 nm and at the two-dimensional graphene is <0.1 eV^54^. We considered carbon vacancies up to six, tested all the possible sites of metal atom binding, and selected the most stable configurations. Representative formation energies (Supplementary Note 4) of single atom configurations and surface adsorption of metal atoms suggest that the metal single atoms prefer to chemically bind with C and O in CNT vacancies, forming stable M–C–O configurations other than being simply adsorbed on CNT (Supplementary Table 2, Supplementary Fig. 23). In addition, the effects of O and N were carefully investigated. As a prototypical system, we investigated Fe doped system, which was found to be the most active and selective for H_2_O_2_ production in the experiments.

It has been shown in the past that the oxygen reduction catalytic activity and selectivity can be related to the binding free energies of reaction intermediates^4,5,27–29^. In the 2e^–^ reduction of O_2_ to H_2_O_2_ (H_2_O_2_ in pH < 11.6 or its deprotonated anion but ${{\mathrm{HO}}_2^ -}$ in pH > 11.6), the catalytic activity can be determined by the binding strength of the key reaction intermediate, *OOH. For the catalysts with weak tendency toward *OOH adsorption, O_2_ activation (Eq. 1) is the potential limiting step (PDS), while desorption of *OOH (Eqs. 2.1 and 2.2) is the PDS for strongly *OOH binding catalysts.

1 $${\mathrm{O}}_2 + {\mathrm{H}}_2{\mathrm{O}} + e^ - \to \ast {\mathrm{OOH}} + {\mathrm{OH}}^ -$$

2.1 $$\ast {\mathrm{OOH}} + e^ - \to \ast + {\mathrm{HO}}_2^ - \;\;\left( {{\mathrm{pH}} > 11.6} \right){\mathrm{or}}$$

2.2 $$\ast {\mathrm{OOH}} + {\mathrm{H}}_2{\mathrm{O}} + e^ - \to \ast + {\mathrm{H}}_2{\mathrm{O}}_2 + {\mathrm{OH}}^ - \left( {{\mathrm{pH}} < 11.6} \right)$$

We therefore use ΔG_*OOH_ as a descriptor to estimate the limiting potential (U_L_) of O_2_ reduction to H_2_O_2_. The maximum *U*_L_, i.e., zero overpotential at the top of the volcano, is 0.68 V vs. RHE originated from the experimental formation free energies of H_2_O_2_. There is a small difference in the formation free energy of its deprotonated anion of ${HO_{2}^ {-}}$, shifting the equilibrium potential positively by 70 mV to 0.75 V vs. RHE in alkaline solutions with pH higher than 11.6. We note that an onset potential around 0.8 V, which is higher than the thermodynamic potential of 0.75 V, was observed in our experimental results. This phenomenon has been reported in previous studies^5,26–28,55^. The main driving force for such difference has been suggested to be the Nernst-related potential shift due to the low concentration of H_2_O_2_ in the electrolyte. We present the volcano plots in the form of H_2_O_2_ with the 0.68 V equilibrium potential throughout this work unless stated otherwise to represent a wider pH range for general mechanism understanding. It should be noted that catalysts positioned at the left leg of the volcano plot are expected to be less selective toward H_2_O_2_. This is due to their strong *OOH adsorption energy, which favors dissociating the O–O bond and driving the 4e^–^ pathway with H_2_O as the major product^5,27–29^. Our results show that in the examined Fe–C systems without O, Fe, and the C atoms around Fe present U_L_ much smaller than 0.68 V and therefore do not contribute to the ORR activity (Supplementary Fig. 24). Once oxygen is included into the systems, most of the C atoms in the vicinity of the Fe–C–O motif (Fig. 4b) show very high activity for H_2_O_2_ production and are positioned near the top of the volcano. The fact that most of the C sites are mainly located at the right leg of the volcano, implies that the C atoms of Fe–C–O motifs could also be selective for 2e^–^ product of H_2_O_2_ over 4e^–^ to H_2_O. This trend remains valid in strong alkaline solutions with the formation of ${HO_{2}^ {-}}$ as shown in Supplementary Fig. 25, as in the theoretical model the difference in the equilibrium potential only results in the change of the peak position by 0.07 eV. In addition, we found out that the incorporation of Fe atoms in Fe–C–O motifs, compared to those with only O dopants, can generally strengthen the *OOH binding on C sites (Supplementary Fig. 26). This as a result significantly improves the H_2_O_2_ activity towards the top of the volcano, suggesting the critical roles of both Fe and O atoms in the coordination. We also calculated the ORR activity of Fe sites in different Fe–C–O model systems, and realized that those Fe sites generally bind the *OOH too strongly, resulting in O–O bond dissociation (Fig. 4b). The calculated *U*_L_s of the Fe in variety of Fe–C–O are <0.3 V, indicating a very low ORR activity. Therefore, we suspect that the C site in Fe–C–O motif is where O_2_ gets selectively reduced to H_2_O_2_. This is in consistent with our *in-Operando* XANES spectra where the oxidation state of Fe remained unshifted, suggesting no additional Fe-O bonding formed during the reaction conditions (Supplementary Fig. 27).

To investigate the effect of local coordination environment on selectivity, we also modeled variety of Fe–C–N configurations, where O atoms are replaced by N (Supplementary Fig. 24c). Our results show that the Fe sites in all the Fe–C–N motifs are rather active for ORR to H_2_O with *OOH adsorption energies positioned in the range of interests for 4e^–^ pathway^4^. This is in a very good agreement with our experimental results as well as previous literature, that Fe–C–N system is highly active for ORR in fuel cell applications^13,56^. Contrary to the Fe–C–O coordination, C sites of Fe–C–N system bind *OOH very weakly (Supplementary Fig. 24c), indicating that C sites are not active for 2e^–^ nor 4e^–^ ORR processes. The Fe active site in Fe-N-CNT was again confirmed by our *in-Operando* XANES experiment that, the oxidation state of Fe was slightly increased due to new Fe-O bonds formed under reaction conditions (Supplementary Fig. 27)^50,57–59^. Additional calculations of different M-C-O motifs in M-CNT catalysts as well as CNT support were presented by volcano plots in Supplementary Fig. 28, where the general trend of H_2_O_2_ selectivity matches with our experimental results.

### Water disinfection by Fe-CNT catalyst

**Fig. 5:**
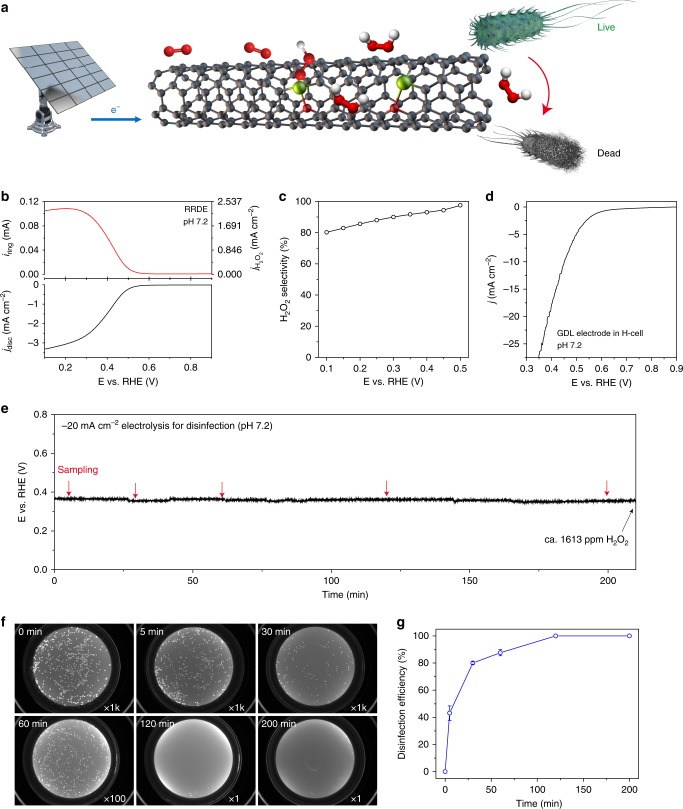
Disinfection performance of Fe-CNT in neutral pH. **a** Schematic of electrochemical synthesis of H_2_O_2_ for water disinfection, with green inputs such as sunlight, air, and water. **b**, **c** LSV of Fe-CNT catalyst on RRDE at 1600 rpm in 0.1 M PBS (pH 7.2) with the corresponding H_2_O_2_ selectivity under different potentials. **d** LSV of Fe-CNT catalyst on GDL electrode (0.5 mg cm^−2^ mass loading) in an H-cell electrolyzer. **e** Bulk electrolysis at a constant current density of 20 mA cm^−2^ in 0.1 M PBS containing ~10^7^ c.f.u. mL^−1^ *E coli* bacteria. The H_2_O_2_ concentration of 1613 ppm at 210 min was determined from a bacteria-free control experiment. **f** CCD photos of overnight cultured plates with spread droplets taken from different time slots during the electrolysis. Dilution factor is labeled at the right bottom corner of each image. No bacteria colonies were observed after 2-h treatment. **g** The disinfection efficiency as a function of treatment time. The error bar represents two identical cultured plates

A wide range of practical applications could be realized with this low-cost Fe-CNT catalyst for highly efficient H_2_O_2_ generation. Since H_2_O_2_ has been widely used in killing bacteria^60^, one promising field is the delocalized or green-route water disinfection with accessible inputs including sunlight for electricity, air for O_2_, and water as shown in the schematic in Fig. 5a. Here we performed a prototype experiment to test the catalyst’s disinfection effectiveness. Neutral pH needs to be used instead of alkaline solutions to mimic the practical applications, therefore the ORR selectivity of Fe-CNT was first evaluated in 0.1 M PBS electrolyte using RRDE as shown in Figs. 5b and 5c. H_2_O_2_ generation starts at ~0.53 V and maintains a high selectivity above 90% from 0.5 to 0.3 V. The practical electrolysis was performed in an H-cell where Fe-CNT catalyst was casted onto a GDL electrode (0.5 mg cm^−2^ catalyst loading), with the catalytic performance plotted in Fig. 5d. The potential to deliver a 20 mA cm^−2^ constant current for H_2_O_2_ generation remained unchanged over the course of electrolysis (Fig. 5e). Around 1613 ppm H_2_O_2_ was generated within 210 min electrolysis as determined by the colorimetric quantification method, representing an average Faradaic Efficiency of 90.8%. With those performance metrics obtained, electrolyte with *Escherichia coli* (*E. coli*) was then used as a model system at a bacteria concentration of ∼10^7^ colony forming units (c.f.u.) mL^−1^. The disinfection process was monitored by picking up several droplets during the 20 mA cm^−2^ chronopotentiometric measurement, followed by serially dilution and spread plating onto LB agar for overnight culture^61,62^. The dark-field CCD images of agar plates with cultured bacteria colony are shown in Fig. 5f, with the calculated killing rate plotted in Fig. 5g. Fe-CNT demonstrates a rapid disinfection efficiency for *E. coli*, delivering a 43% bacteria inactivation in 5 min and more than 99.9999% in 120 min (equals to a 125 L h^−1^ m^2^_electrode_ processing rate) with no recovery observed (Supplementary Fig. 32). We further suspended *E coli* bacteria into 2-h electrolyzed 0.1 M PBS solution (ca. 1000 ppm of generated H_2_O_2_ concentration) with the image of overnight cultured plate shown in Supplementary Fig. 29, which clearly excludes any effects of disinfection from applied potential. This device can be further upgraded into a flow cell with tunable water flow rate and current density for a well-balanced water disinfection rate and efficiency for specific applications in the future.

## Discussion

Our experimental and theoretical results highlight that the TM single atom coordination motifs can effectively tune the ORR pathways and product selectivity. Among different catalysts examined, Fe–C–O coordination was identified as highly active and selective motif for O_2_ reduction to H_2_O_2_. Given the many choices of TM atoms, supports, as well as non-metal dopants, this wide range of coordination combinations could enrich the pool of catalytic active sites for improving, controlling, and understanding catalytic reactions beyond ORR. Future efforts also could be focused on electrolysis cell design for pure H_2_O_2_ solution generation without solute mixtures. In addition, by integrating renewable electricity from solar or wind with water and air, our earth-abundant Fe-CNT H_2_O_2_ catalyst could therefore pave a green way, different from the traditional anthraquinone process, for H_2_O_2_ generation, water treatment, chemical synthesis, and many other important applications in the future.

## Methods

### Material synthesis and characterization

M-CNT catalysts were prepared by the impregnation and reduction method. In a typical synthesis of Fe-CNT, a 7.5-mM iron nitrate stock solution was first prepared by dissolving Fe(NO_3_)_3_·9H_2_O (ACS Grade, Alfa Aesar) into Millipore water (18.2 MΩ·cm). A carbon suspension was prepared by mixing 50 mg multi-walled carbon nanotubes (Carbon Nanotubes Plus GCM389, used as received) with 20 mL of Millipore water, and tip sonicated (Branson Digital Sonifier) for 30 min till a homogeneous dispersion. Then 200 µL of Fe^2+^ solution, given a raw atomic ratio of Fe:C to be ~0.1 at.%, was dropwise added into CNT solution under vigorous stirring, followed by a quickly frozen in liquid nitrogen. The as-prepared Fe(NO_3_)_3_/CNT powder was heated up in a tube furnace to 600 °C at a pressure of 1 Tor and a gas flow of 100 sccm Ar (UHP, Airgas) within 20 min, and kept at same temperature for another 40 min before cooling down to room temperature. Other Pd-, Co-, and Mn-CNTs were prepared in a similar way to Fe-CNT except for various metal salt precursors, i.e., Pd(NO_3_)_2_·2H_2_O, Co(NO_3_)_2_·6H_2_O, and Mn(NO_3_)_2_·4H_2_O (Puriss or ACS Grade, Sigma-Aldrich), respectively. N doped Fe-N-CNT was prepared by heating up the above-mentioned Fe(NO_3_)_3_/CNT powder under a same temperature program with Fe-CNT but within a mixed gas flow of 50 sccm NH_3_ (anhydrous, Airgas) + 100 sccm Ar.

Probe-corrected FEI Titan Themis 300 S/TEM with ChemiSTEM technology was used for S/TEM characterization. Drift correction was applied during acquisition. SEM was performed on a Zeiss Supra55VP field emission scanning electron microscope with in-lens detector. X-ray photoelectron spectroscopy was obtained with a Thermo Scientific K-Alpha ESCA spectrometer, using a monochromatic Al Kα radiation (1486.6 eV) and a low energy flood gun as neutralizer. Thermo Avantage V5 program were employed for surface componential content analysis.

Ex situ XAS spectra on Co, Mn K-edge were acquired at the SXRMB beamline of Canadian Light Source using a 4-element Si drift fluorescence detector, and Pd K-edge spectra were acquired in fluorescence mode with a 32-element Ge detector at the HXMA beamline. The powder sample was spread onto doublesided, conducting carbon tape. In situ electrochemical XAS measurement on Fe K-edge was carried at Beamline 8-ID, National Synchrotron Light Source II, Brookhaven National Laboratory, using a Si(111) monochromator and a Lytle detector. For in situ spectroelectrochemical tests, continuous O_2_ flow was delivered into a homemade Teflon H-cell filled with O_2_-saturated 0.1 M KOH, a Kapton film covered gas diffusion layer (Fe-CNT/GDL) working electrode was served as the X-ray window for synchrotron radiation. Analyses of both the near edge (in energy scale) and extended range (in *R* space) XAS spectra were performed using Athena software.

### Nanofabrication and APT characterization

Specimens for the 3D Atom Probe Tomography were prepared using an FEI Helios 660 Nanolab Dual-Beam FIB/SEM. Liftout was performed following standard APT sample prep procedures; sharpening of the tips was performed first with an ion beam accelerating voltage of 30 kV, and then (for final shaping and cleanup) at 2 kV, to produce tips with apices of ~30 nm diameter. 3D APT was performed using a Cameca LEAP 4000 HR instrument in laser pulsing mode, with the following operating conditions: laser pulse energy of 100 pJ and repetition rate of 100 kHz; base temperature of 40 K; detection rate of 0.5%. The dataset shown in the main text consisted of slightly more than 9M ions, of which the majority were Ni from the capping layer above the Au-CNT-Au sandwich. No specimens survived to show the lower Au layer, likely due to the high porosity of the CNT powder material. Analysis of that data was performed using Cameca IVAS 3.16.4 software, with reconstructions based on SEM images of the tip profile. Mass spectrum peaks were ranged using the full-width-at-one-tenth-maximum method. Only one peak was ranged as Fe, that being the peak at 56 Da; no other Fe isotopes could clearly be identified both due to mass overlaps with other species and due to the low number of detected atoms in this peak (sufficiently low that the other Fe isotopes would be expected to be undetectable below the background noise even without mass overlaps). This implies that the stated conclusions about Fe in this system, as determined by APT, represent an upper limit on the possible number and distribution of Fe atoms around the CNT.

### Electrochemical measurements

A BioLogic VMP3 work station was employed to record the electrochemical response. Certain amounts of KOH (Reagent Grade, Sigma-Aldrich) or K_2_HPO_4_/KH_2_PO_4_ (ACS Grade, Sigma-Aldrich) was dissolved in Millipore water to prepare the 0.1 M electrolyte. The rotation ring disk electrode (RRDE) measurements were run at 25 °C in a typical three-electrode cell. A platinum foil (99.99%, Beantown Chemical) and a saturated calomel electrode (SCE, CH Instruments) were used as the counter and reference electrode, respectively. A RRDE assembly (AFE6R1PTPK, Pine Instruments) consisting of a glassy carbon rotation disk electrode (*Φ* = 5.0 mm) and a Pt ring (*Φ* = 15.0 mm) was used, with a theoretical collection efficiency of 25%. Experimentally, the apparent collection efficiency (*N*) was determined to be 24.1% in the ferrocyanide/ferricyanide half reaction system at a rotation rate between 400 and 2025 rpm (Supplementary Fig. 9)^63^. To prepare M-CNT cast working electrode, typically, 3.3 mg of as-prepared M-CNT catalyst was mixed with 1 mL of ethanol and 10 µL of Nafion 117 solution (5 %, Sigma-Aldrich), and sonicated for 20 min to get a homogeneous catalyst ink. 6 µL of the ink was pipetted onto glassy carbon disk (0.196 cm^2^ area, 0.1 mg/cm^2^ mass loading), got vacuum dried prior to usage. As the catalyst can be dispersed very well in ethanol solutions, uniform catalyst coating can be made on the disc electrode without obvious pin holes or uncovered edge. All potentials measured against SCE was converted to the reversible hydrogen electrode (RHE) scale in this work using E (vs. RHE) = E (vs. SCE) + 0.244 V + 0.059*pH, where pH values of electrolytes were determined by Orion 320 PerpHecT LogR Meter (Thermo Scientific, i.e., 13.0 for 0.1 M KOH and 13.9 for 1 M KOH). This SCE reference electrode was further calibrated to freshly prepared RHE prior to usage (Supplementary Fig. 13), in good agreement with the calculated values. The diffusion limited current of our catalyst is at around 3 mA cm^−2^ and is higher than that of bare glassy carbon, which may due to the low density of active sites on mirror polished glassy carbon electrode. A gradual degradation of ring current was observed during the continuous RRDE stability test, which was mainly due to the surface oxidation of Pt ring electrode constantly operated at high potential (1.2 V, Fig. 3c) and can be readily recovered by rapid cyclic voltammetry at low potentials to reduce PtO_x_. H_2_O_2_ selectivity was calculated using the following equation: H_2_O_2_ (%)=$200 \times \frac{{I_{{\mathrm{Ring}}}/N}}{{I_{{\mathrm{Disk}}} + I_{{\mathrm{Ring}}}/N}}$, and the electron transfer number (*n*) at the disk electrode during ORR was calculated using $n = \frac{{4\left| {I_{{\mathrm{Disk}}}} \right|}}{{I_{{\mathrm{Disk}}} + I_{{\mathrm{Ring}}}/N}}$, where *I*_Ring_ is the ring current, *I*_Disk_ is the disk current and *N* is the collection efficiency.

Bulk H_2_O_2_ production in 1 M KOH was carried out in a customized H-cell electrolyzer, with 0.5 mg cm^−2^ Fe-CNT air-brushed onto a 1×2.5 cm^2^ Freudenberg GDL electrode (Fuel Cell Store) as ORR cathode, with the anode of 0.2 mg cm^−2^ IrO_2_/GDL for water oxidation. A Fumasep FAA-3-PK-130 anion exchange membrane (Fuel Cell Store) was employed to separate the chambers. H_2_O_2_ concentration was quantified by cerium sulfate titration based colorimetric method (2Ce^4+^ + H_2_O_2_ → 2Ce^3+^ + 2 H^+^ + O_2_)^27^. The H_2_O_2_ concentration-absorbance curve was calibrated by mixing known amount of commercial H_2_O_2_ solution with 1 mM Ce(SO_4_)_2_. The absorption at 320 nm wavelength was measured on a Cary 5000 UV-Vis-NIR spectrometer (Agilent) and used to determine the Ce^4+^/H_2_O_2_ concentration (Supplementary Fig. 18). To fit the linear range of calibration curve, the electrolyte collected was further diluted by 10 to 100 times in 0.5 M H_2_SO_4_.

### Computational details

Density functional theory calculations were performed using the Vienna Ab Initio Simulation Package (VASP)^64,65^. We used BEEF-vdW exchange-correlation functional, which has been shown to accurately describe chemical and physical interactions between adsorbates and graphene^66^. We set an energy cutoff, a convergence criteria for self-consistent iteration and ionic relaxation to be 500 eV, 10^−4^ eV and 0.05 eV/Å, respectively. Bulk graphene unit cell was optimized using (12 × 12 × 1) k-points mesh, resulting in C-C distance to be 1.424 Å. (7 × 7) supercell consisting of 98 carbon atoms with 15 Å of a vacuum perpendicular to the graphene plane was employed to model carbon materials, and we considered up to six carbon vacancies. Various sites for a single metal atom adsorption were assessed, and the effects of oxygen or nitrogen near the single metal atom were taken into account as well. For the adsorption calculations, (2 × 2 × 1) k-points mesh was utilized.

We analyzed *OOH adsorption on the catalysts, where the possible catalytic active site is either the single metal or nearby carbon sites. For single atom catalysts, the single metal atom is significantly under-coordinated compared to their bulk counterparts resulting in markedly strong binding of adsorbates, and it is highly likely that the site is pre-occupied by other adsorbates, such as *O and *OH, under the ORR conditions. In this sense, for metal site adsorptions, we first determined the most relevant coverage of the metal atom at 0.7 V_RHE_ by constructing a surface Pourbaix diagram (Supplementary Figs. 29-31). For carbon site adsorptions, we considered all carbon sites near the single metal atom and reported the most stable binding free energies.

To construct a free energy diagram of ORR to H_2_O_2_, we corrected the calculated electronic energies by adding zero-point energy, enthalpy and entropy of adsorbate at 300 K obtained from a harmonic oscillator approximation using Atomic Simulation Environment^67^. Since O_2_ molecule is poorly described by standard DFT calculations, we used the calculated energies of gas-phase H_2_O and H_2_ molecules, and experimental formation free energies of H_2_O and H_2_O_2_ as references. To take into account the effect of the electrode potential, computational hydrogen electrode (CHE) method was employed^68^. In this method the chemical potential of proton and electron pair *μ*(*H*^+^+*e*^–^) is equal to one half of that of gas-phase hydrogen molecule $(1/2\mu (H_2))$ at standard conditions, and the effect of the electrode potential is included by shifting the electron free energy by −*eU*_elec_, where *e* and *U*_elec_ are elementary charge and electrode potential, respectively.

### Water disinfection

A standard lab bacterial Escherichia coli strain was kindly provided by Howard Berg’s laboratory. *E. coli* was cultured to stationary phase in LB broth for 14 h at 37 °C, harvested by centrifugation at 800 × *g*, washed three times with 0.1 M PBS solution and suspended in 0.1 M PBS to ~7.1 × 10^6^ c.f.u. mL^−1^. The electrochemical disinfection measurements were run at 25 °C in a H-type glass cell separated by a Fumasep FBM bipolar membrane (Fuel Cell Store). 25 mL of the prepared E. Coli in 0.1 M PBS was injected into the cathodic chamber, with a 1 cm^2^ Fe-CNT/GDL (0.5 mg cm^−2^ catalyst loading) serving as the working electrode. A chrono-potentiometric measurement at a fixed current density of 20 mA cm^−2^ was performed to ascertain water disinfection. Another control experiment was run in 25 mL of bacteria-free electrolyte to calculate the overall H_2_O_2_ concentration and generation rate. The pH value was noted to maintain ~7.2 before and after the continuous electrolysis in PBS buffer. Bacterial concentrations and killing rates were measured at different time points during electrolysis using standard spread plating techniques. Each sample was serially diluted and each dilution was plated in triplicate onto LB agar plates, and incubated at 37 °C for 12 h. The images of overnight cultured plates were taken with a custom-built dark-field imager equipped with a CCD camera (Point Grey Chameleon).

## Supplementary information

Supplementary Information

Peer Review File

Description of Additional Supplementary Files

Supplementary Movie 1

## Data Availability

The data that support the findings of this study are available from the corresponding authors upon reasonable request.
