# Supplementary material for: Highly selective oxygen reduction to hydrogen peroxide on transition metal single atom coordination
Source: Nat Commun. 2019 Sep 5;10:3997. doi: 10.1038/s41467-019-11992-2 (PMC6728328; doi:10.1038/s41467-019-11992-2)
Supplement: Supplementary file 1 — Supplementary Information [file 41467_2019_11992_MOESM1_ESM.pdf]

Supplementary Information for

Highly selective oxygen reduction to hydrogen peroxide on transition metal single atom coordination

Jiang et al.

Supplementary Note 1. Metal atom loading and acid stability

Earlier reports of Fe-N-C in literature, or other transition metal single atom catalysts, usually start with an excessive Fe doping in the Fe-C precursor, and thereafter an acid leaching was employed to get rid of free Fe nanoparticles and leave with single Fe atoms coordinated in graphene vacancies for high catalytic performance. However, acid leaching method does not apply in our case of Fe-CNT catalyst, as we observed that single atomic Fe-C-O motifs were not stable in acids. We performed acid leaching test by sonicating the Fe-CNT catalyst in 10 mL of 1 M $HClO_4$ for 1 h, with Fe ion contents in the $HClO_4$ solution quantified by ICP-MS (Agilent 7900, with a detection limit of ~1 ppb for Fe). Different from the acid resistive feature observed in Fe-C-N motif, nearly all of the Fe atoms (ca. 92%) from Fe-C-O motifs were leached out into the $HClO_4$ solution, suggesting that the Fe-C-O motifs are not stable in strong acids. Complicated studies and mechanisms are needed to explain this different stability of Fe-C-N and Fe-C-O motifs in acids, but we suspect that this might be related with the similarly different acid-resistance between iron nitrides and oxides. Therefore, instead of using acid leaching method, our strategy is to only include a small amount of Fe precursors to ensure a uniform and atomic dispersion as demonstrated by our XAS, TEM, and APT experiments. As a control synthesis, when we double the amount of Fe loading from current 0.1 to 0.2 at.%, we observed the possible formation of iron oxide clusters in addition to Fe single atoms as shown in the EXAFS spectrum of Supplementary Fig. 2. Therefore, we selected the 0.1 at.% mass loading to ensure atomic dispersion of Fe atoms.

On the other hand, clusters instead of single atoms formed when we reduce the density of CNT defects/vacancies but maintain the Fe atom loading. As shown in Supplementary Fig. 3, the I_D/I_G ratio of mildly oxidized CNT (1-h activation at 60 °C within 12 M nitric acid) is lower than the CNT-COOH we used in our Fe-CNT sample, which resulted in the formation of Fe oxide clusters shown by EXAFS in Supplementary Fig. 2.

Supplementary Note 2. Oxidation states of coordinated and simply adsorbed Fe on CNT

For a fair comparison, the simply adsorbed Fe sample was prepared in the same way with Fe-C-O except for the 600℃ annealing process during which Fe atoms can form coordination with C and O. As shown in the Fe K-edge XANES of Supplementary Fig. 10, the oxidation state of

coordinated Fe is lower than the simply adsorbed Fe (which is Fe^{3+} ions from $Fe(NO_3)_3$ precursor), confirming the different chemical environment between the adsorption case and coordination case. This result provides another support to our conclusion of the formation of Fe-C-O coordination.

Supplementary Note 3. Analysis of possible H_2O_2 decomposition on metal oxides

We understand that MnO_x could quickly decompose H_2O_2 species as reported by previous literatures which may affect the quantification of H_2O_2 using ring-disc setup. Here we can exclude this possibility by the following two aspects:

First, we can do a simple order-of-magnitude estimation of the H_2O_2 decomposition rate compared with generation rate. The decomposition rate can be written as $-\frac{d[H_2O_2]}{dt} = k[MnOx][H_2O_2]$. From literature we understand that the H_2O_2 decomposition rate constant (k) on manganese oxide is on the order of 1 M^{-1} s^{-1} (we select the highest number from Supplementary Ref. [1]. The concentration of manganese oxide can be estimated by the catalyst mass loading density of 0.1 mg cm^{-2} and the catalyst layer thickness. We do not know the exact catalyst thickness but we can estimate a minimal value to ensure a maximal estimation of decomposition rate, therefore a very high CNT tap density of about 1 g cm^{-3} was used for a thickness of 10^{-4} cm (significantly underestimated). We therefore get a lowest estimation of Mn concentration of ~ 0.05 M, which gives a decomposition rate of 0.05 [H_2O_2] s^{-1} ([H_2O_2] represents the generated H_2O_2 concentration). Since the RRDE test was under 1600 rmp rotation where generated H_2O_2 can be quickly diffused to Pt ring by the forced convection (at least one order of magnitude shorter than 1 s), and even we overestimate the diffusion time to be ~ 1 s, the decomposition was only about 5% of the generated H_2O_2. We therefore believe that the H_2O_2 decomposition plays a negligible role in quantifying H_2O_2 selectivity in our RRDE testing system.

Secondly, other metal oxides including Fe or Pd also present similar H_2O_2 decomposition rates compared with Mn[2], but we still see the difference in H_2O_2 selectivity. In addition, the Fe-CNT H_2O_2 selectivity quantified by RRDE and cerium sulfate titration based colorimetric method agrees very well, further excluding the decomposition effects. We therefore believe that the activity order in selective O_2 reduction to H_2O_2 is more of intrinsic activity difference on M-C-O motifs rather than H_2O_2 decomposition.

Supplementary Note 4. Formation energies analysis of Fe-C-O vs. surface adsorbed Fe/CNT

We calculated formation energies of two representative Fe single atom configurations (5V-O_2 and 5V-O_1) located on the top region of the volcano plot (Supplementary Table 2 and Supplementary Fig. 23). The formation energies were calculated as follows: E[total] – E[support] – E[Fe], where E[total] and E[support] are total energies of Fe atom and support complex, and support itself, respectively. To calculated E[Fe], we used a body centered cubic bulk Fe as a reference. Therefore, the calculated formation energies predict the relative stability of the single atom configuration or surface adsorption against Fe clustering into bulk Fe. Note that the Fe atom adsorbed on the CNT surface site was relaxed to the lowest energy for its most stable configuration. As shown in the Supplementary Table 2, the formation energies of the Fe-C-O single atom configurations are always more negative compared to the surface adsorption. In addition, we show that the formation of the single atom configurations is more favorable than forming bulk Fe clusters (energy less than zero), suggesting their strong driving force towards the single atom configurations, and thus high structure stability. The results show similar trends to the case of Fe-N-C motifs with representatives listed in the Table. We note that a relaxation of the surface adsorption of N-decorated supports resulted in the formation of the single atom configuration, indicating that the Fe adsorption on the surface is highly unlikely. Therefore, we believe it is most likely that Fe single atoms prefer to chemically bind with C and O in CNT vacancies, forming stable Fe-C-O configurations other than being adsorbed on CNT. We further calculated the in-plane atomic structures of 5V-O_1 and 5V-N_3 in the presence of a water monolayer (Supplementary Fig. 23c), as model cases for Fe-C-O and Fe-C-N coordination as listed in the formation energy table in point 1 in our previous response letter. The water monolayer is taken from Supplementary Ref [3], which studied a similar single atom catalyst supported on two-dimensional materials. A solvation stabilization energy was calculated as E[total+water] - E[water] - E[total], where E[total+water] and E[water] is total energies of Fe atom, support complex and the water monolayer, and the water monolayer itself, respectively. The calculated solvation stabilization energy was -3.11 eV, indicating that the Fe-C-O 5V-O_1 motif will be further stabilized in an aqueous condition. Fe-C-N shows the similar trend with a further stabilization energy of -1.58 eV.

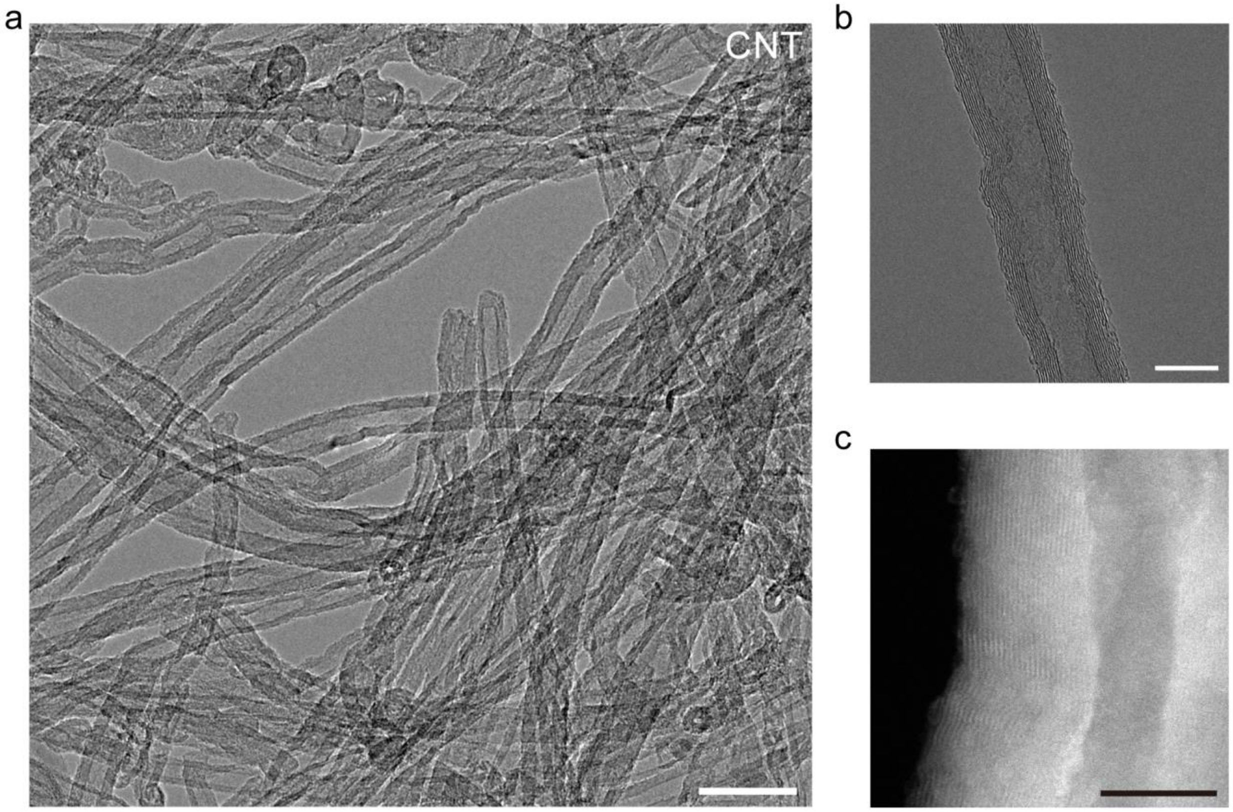

Supplementary Figure 1. Characterization of CNT substrates. (**a**) large area TEM image, (**b**) zoom-in high resolution TEM image and (**c**) Aberration corrected HAADF-STEM image of a single carbon nanotube. The bare CNT shows clearly rolled graphene layers, but no bright dots were observed as compared to the metal doped ones in Fig. 1. Scale bars, 50 nm in (**a**), 10 nm in (**b**), 5 nm in (**c**).

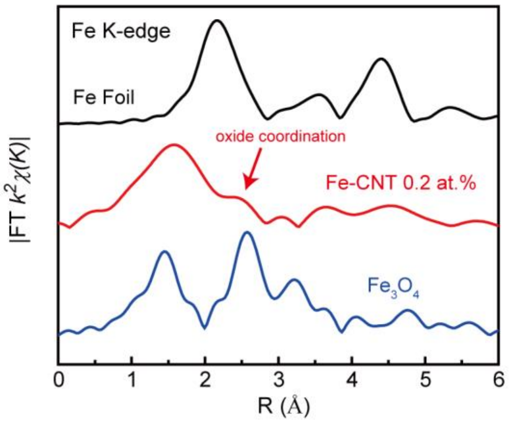

Supplementary Figure 2. Fe K-edge EXAFS of Fe-CNT catalyst with doubled Fe loading. Possible formation of iron oxide clusters is observed.

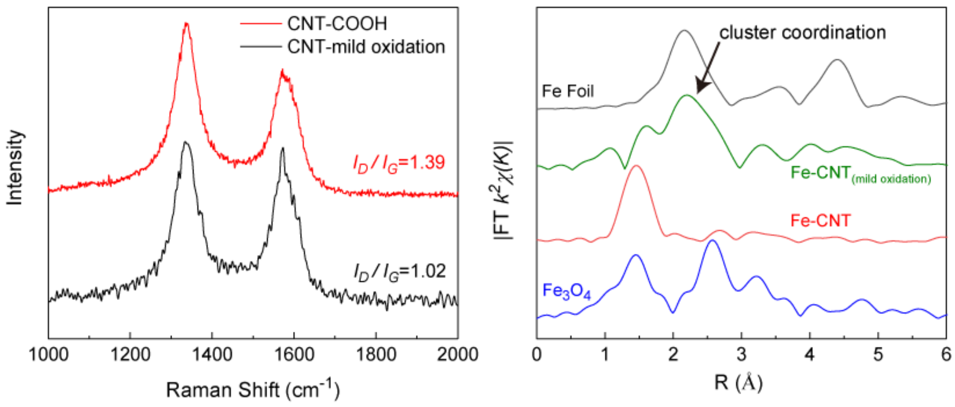

Supplementary Figure 3. Raman and EXAFS characterizations of Fe-CNT. The I_D/I_G ratio in Raman spectra suggest the different density of vacancies/defects,. the less disordered CNT support showed formation of clusters with the same Fe atom loading.

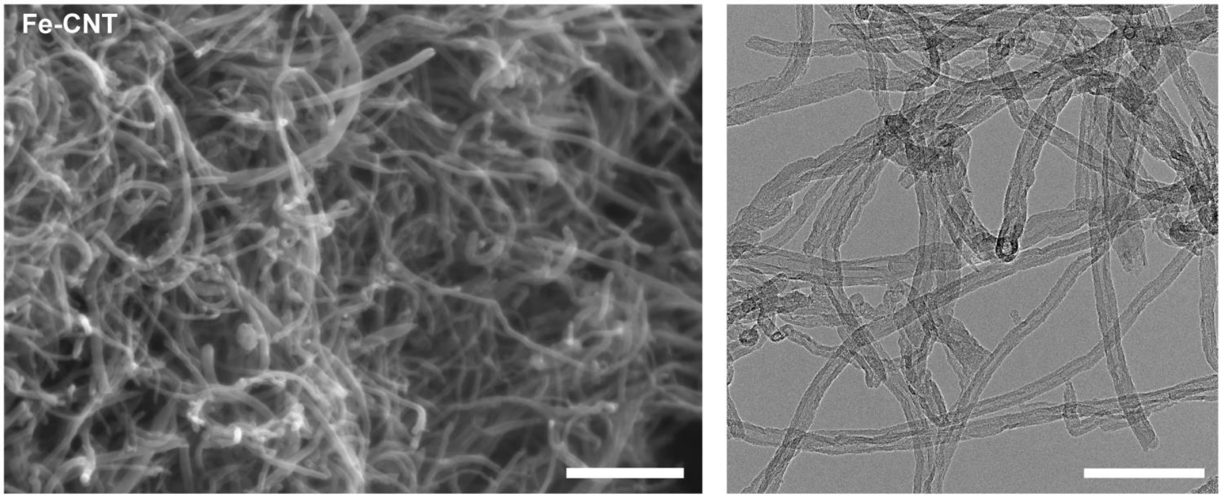

Supplementary Figure 4. SEM and large scale TEM images of Fe-CNT. Scale bars: 200 nm (left) and 100 nm (right).

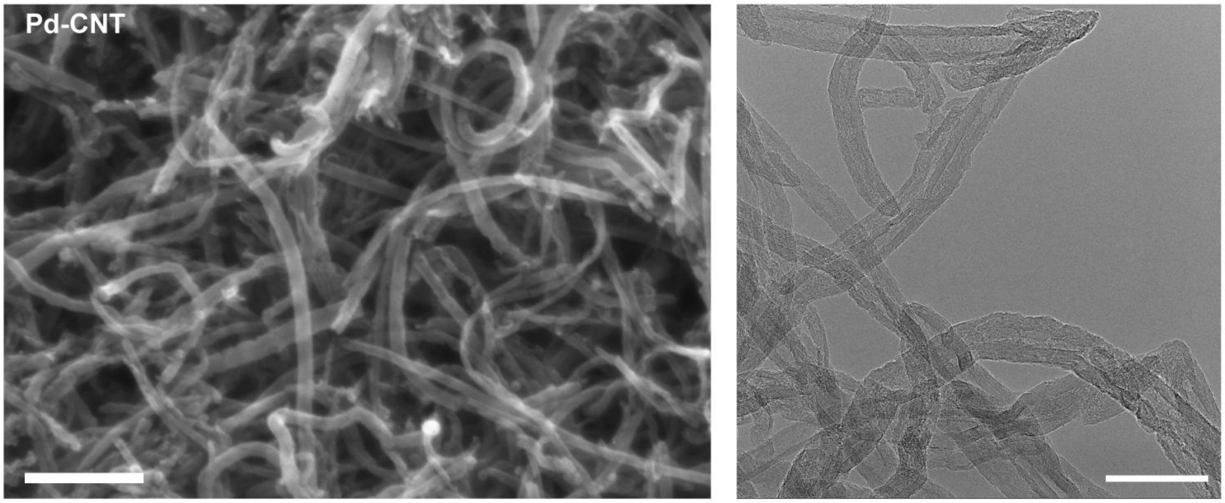

Supplementary Figure 5. SEM and large scale TEM images of Pd-CNT. Scale bars: 200 nm (left) and 50 nm (right).

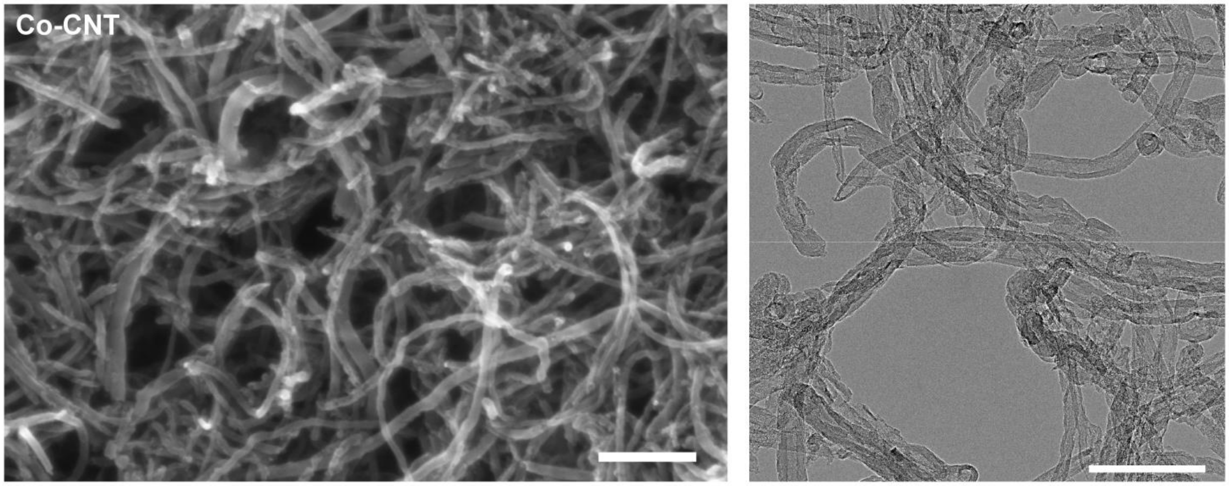

Supplementary Figure 6. SEM and large scale TEM images of Co-CNT. Scale bars: 200 nm (left) and 100 nm (right).

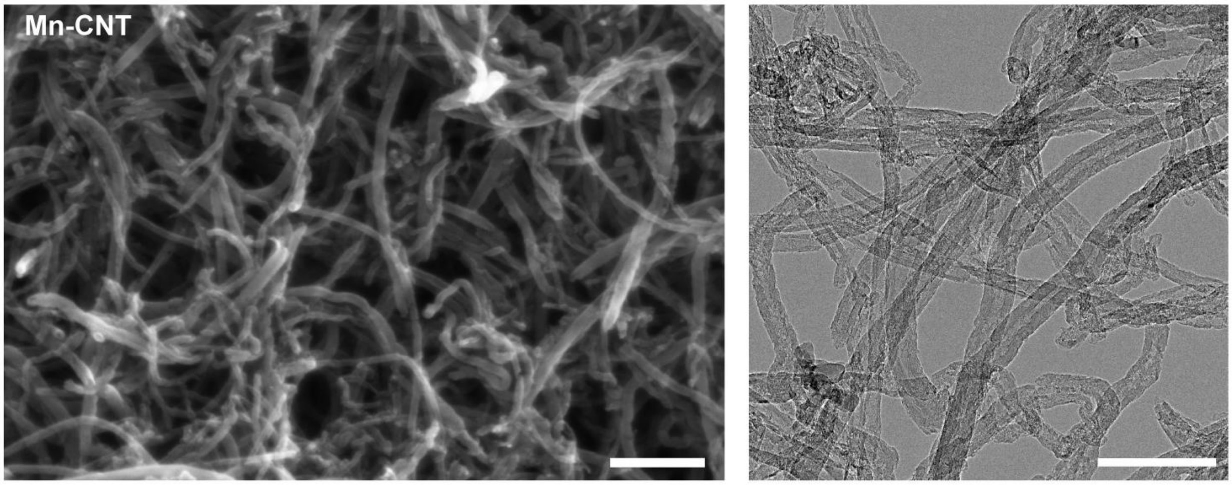

Supplementary Figure 7. SEM and large scale TEM images of Mn-CNT. Scale bars: 200 nm (left) and 100 nm (right).

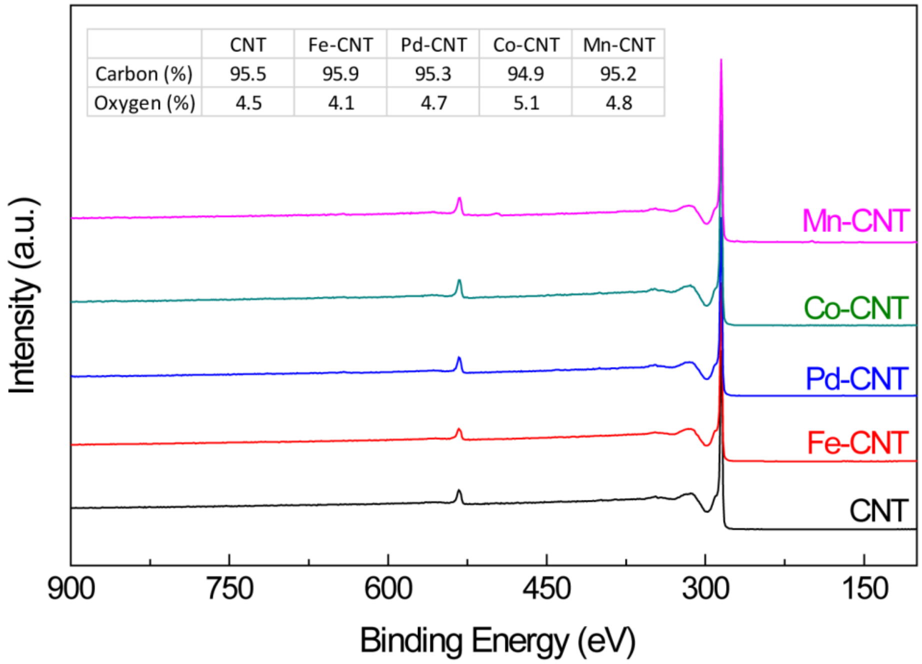

Supplementary Figure 8. XPS survey spectra of bare CNT and Fe-, Pd-, Co-, Mn-doped CNT. Only C (285.1 eV) and O (532.8 eV) elements are detected and insert is their atomic content table. Similar C:O ratios were observed on these samples, ruling out the oxygen content effect on the H_2O_2 selectivity.

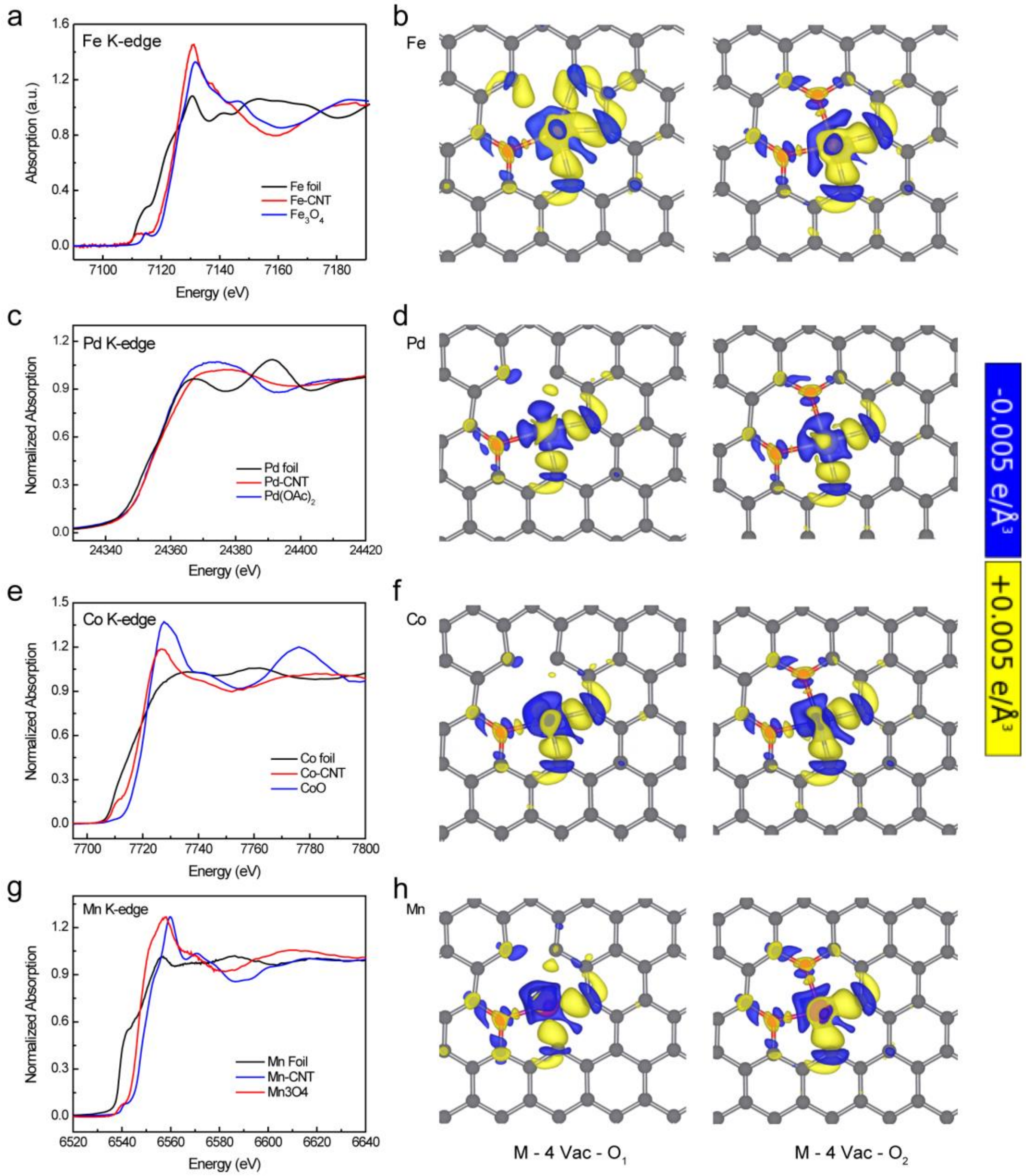

Supplementary Figure 9. Near edge XANES spectra and corresponding charge density plot upon metal atom adsorption ($\Delta\rho = \rho_{Gr+M} - \rho_{Gr} - \rho_M$) for isolated metal atom doped CNTs. (**a**, **b**) for Fe-CNT, (**c**, **d**) for Pd-CNT, (**e**, **f**) for Co-CNT and (**g**, **h**) for Mn-CNT. Blue and yellow represent electron depletion and accumulation, respectively. The pronounced delocalization observed in both charge density difference plots indicates a strong interaction and a significant charge transfer from single metal atoms to coordinated C or N atoms.

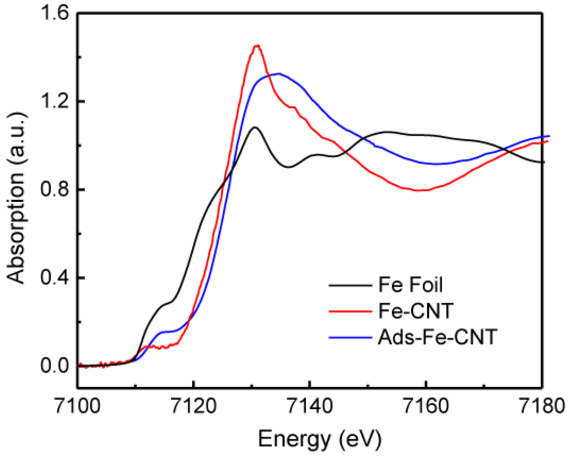

Supplementary Figure 10. Fe K-edge XANES spectra comparison. The oxidation state of coordinated Fe is lower than the simply adsorbed one.

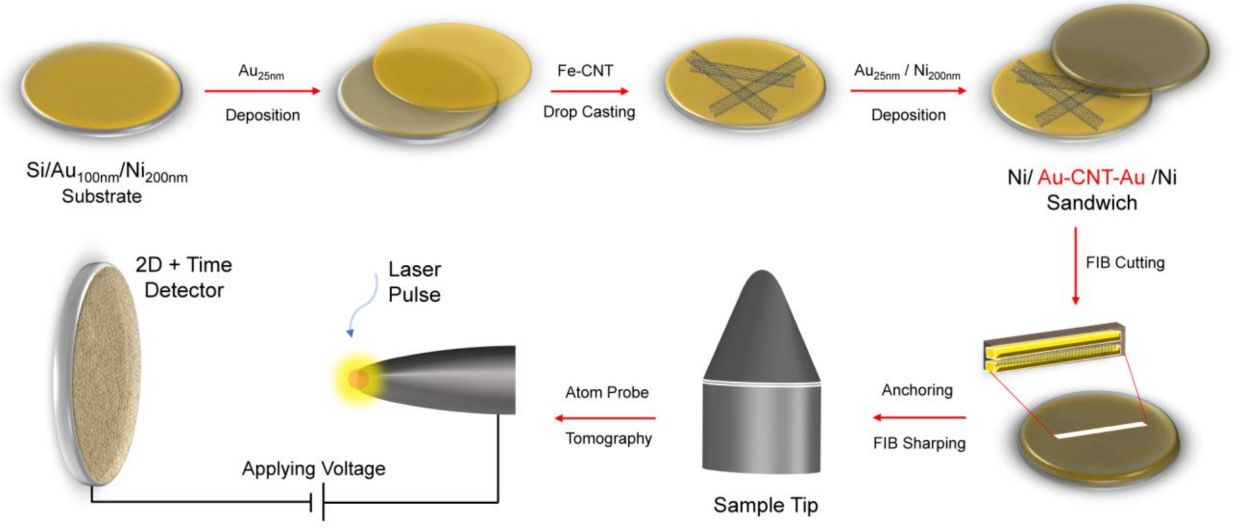

Supplementary Figure 11. Nanofabrication procedures to prepare APT working tips.

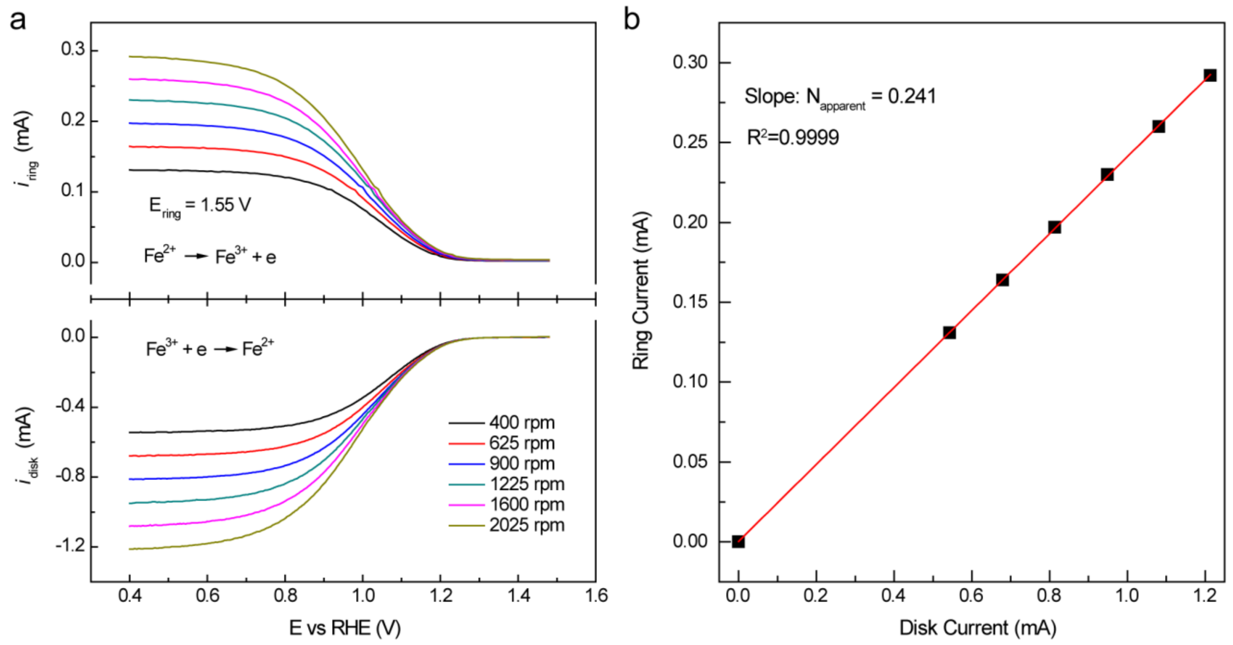

Supplementary Figure 12. RRDE collection efficiency calibration. (**a**) Linear sweep voltammetry curves recorded on a bare glassy carbon rotation disk electrode (Φ = 5.0 mm) with a Pt ring (Φ = 15.0 mm) in 0.1 M KOH supporting electrolyte with 10 mM $K_3Fe(CN)_6$. Sweep rates: 20 mV s^{-1}, E_{ring} = 1.55 V vs. RHE. (**b**) Linear fitting of the diffusion limited current densities recorded on ring and disk electrodes at different rotation speed. The experimental determined apparent collection efficiency (N) is 24.1%, close to the theoretical vale of 25.0% (Pine AFE6R1PTPK).

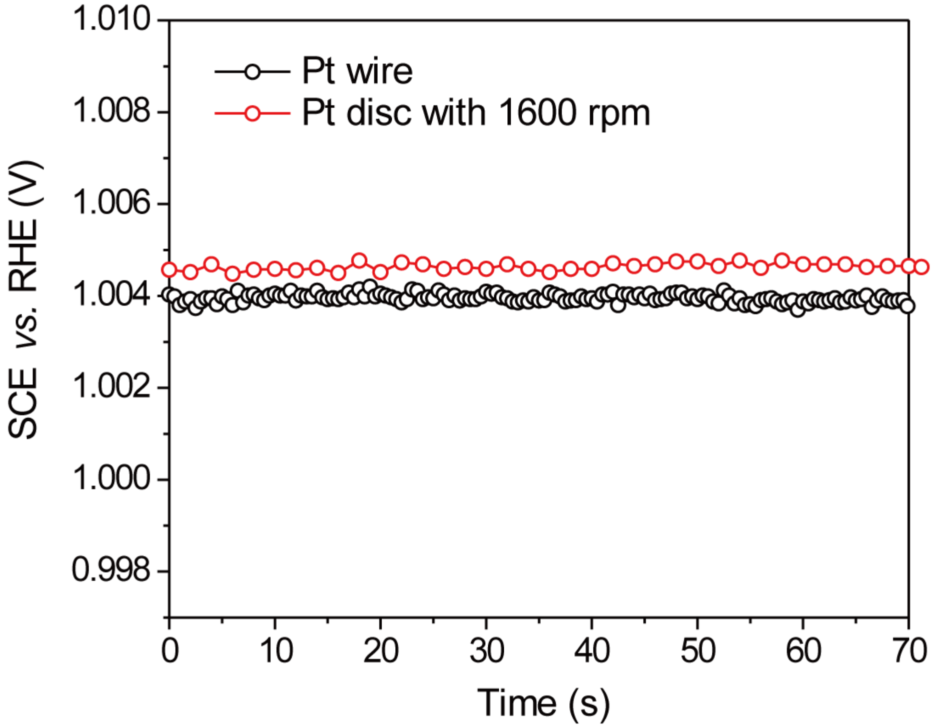

Supplementary Figure 13. Reference electrode calibration. Potential calibration of the SCE reference electrode to RHE scale by purging pure H_2 gas onto a physically and electrochemically polished polycrystalline Pt wire or Pt rotation disc electrode at a reasonable rotation speed in H_2-saturated 0.1 M KOH electrolyte. The calibrated potential is 1.004 ~ 1.005 V *vs.* RHE, in a good agreement of the calculated potential of 1.010 V *vs.* RHE.

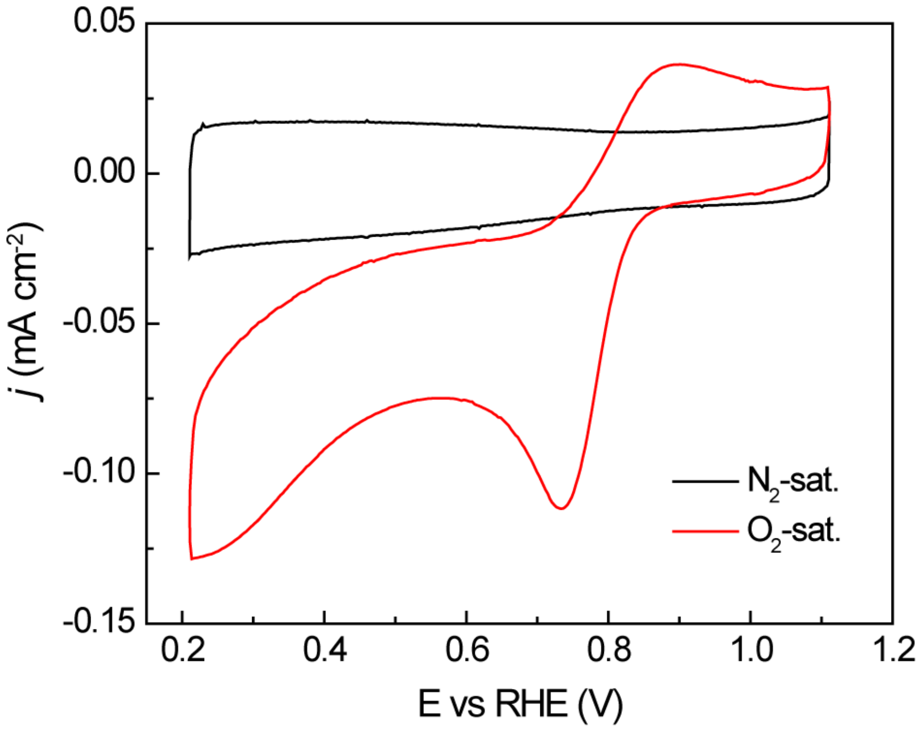

Supplementary Figure 14. Cyclic voltammograms of Fe-CNT cast glassy carbon electrode in 0.1 M KOH saturated with N_2 and O_2, respectively. No redox feature of Fe but only the double layer current of CNT substrate was observed in N_2-saturated electrolyte, suggestive the absence of any Fe nanoparticles in the catalyst. The oxidation peak occurred in O_2-saturated electrolyte was probably arisen from the oxidation of *OOH, HO_2^-, or other intermediate species, which were generated and adsorbed on the Fe-CNT catalyst surface at low potential regions during the negative CV scanning. Since we are testing the CV without rotation, those generated products or intermediates may not be quickly diffused away and then got oxidized in the positive-going potential sweep. Catalyst loading: 0.1 mg cm^{-2}, sweep rates: 20 mV s^{-1}.

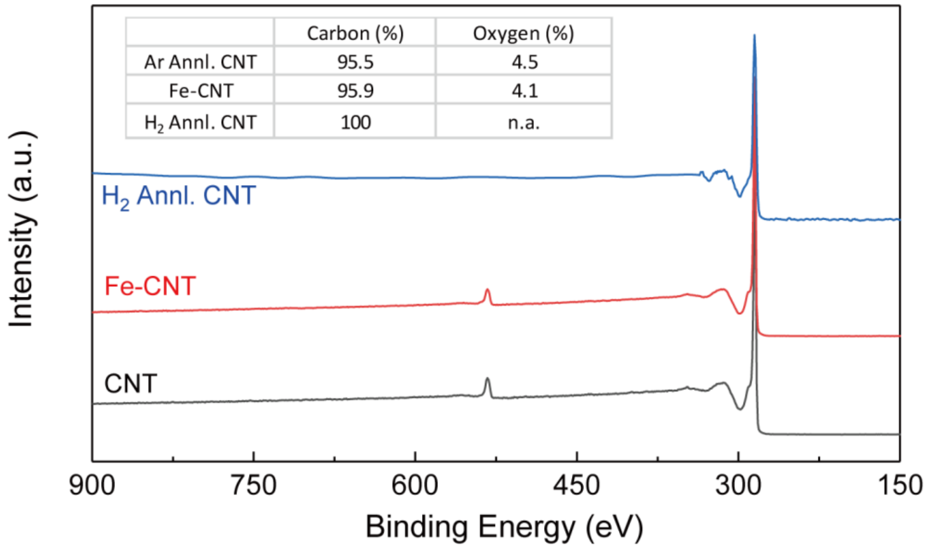

Supplementary Figure 15. XPS survey spectra of Ar-annealed CNT (black), Fe-CNT (red) and H_2-annealed CNT (blue). Since our Thermo Scientific K-Alpha XPS apparatus does not include an in-situ high-temperature degas capability, to maximally avoid air contamination, our experimental procedure is to quickly transfer the freshly synthesized catalyst (under 600 ℃ annealing) into an Ar glove box for XPS sample preparations and then seal the sample by air tight pouches during the sample transportation to the XPS machine. While there were still inevitable air exposures, we believe the effects became negligible as demonstrated by a control experiment we performed as a side-proof. We prepared a H_2 annealed CNT sample (the same CNT we used as our catalysts' support) to get rid of its surface oxygen. Following exactly the same XPS sample preparation procedures, we did not observe any detectable O signals as shown in the figure, suggesting that there are negligible O signal contributions from the potentially adsorbed CO_2 or O_2 from the air.

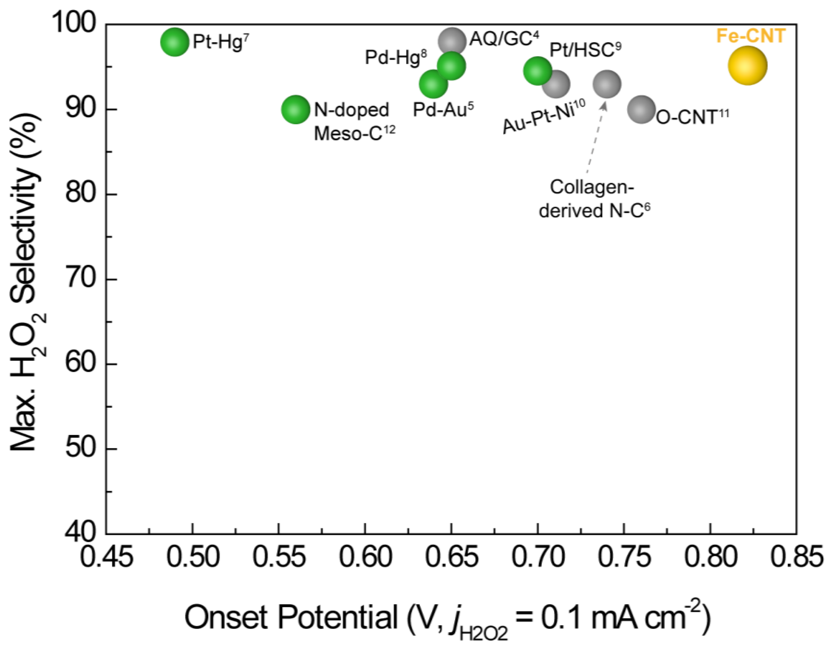

Supplementary Figure 16. H_2O_2 generation performance comparison. RRDE performance map of maximum H_2O_2 selectivity and onset potential (defined as the potential delivering 0.1 mA cm^{-2} H_2O_2 partial current) of reported electrocatalysts[4-12]. Green and grey balls represent acidic and alkaline electrolyte, respectively. Please be noted that the equilibrium potential in strong alkaline electrolyte (pH > 11.6) is 70 mV more positive than that in neutral pH or acids.

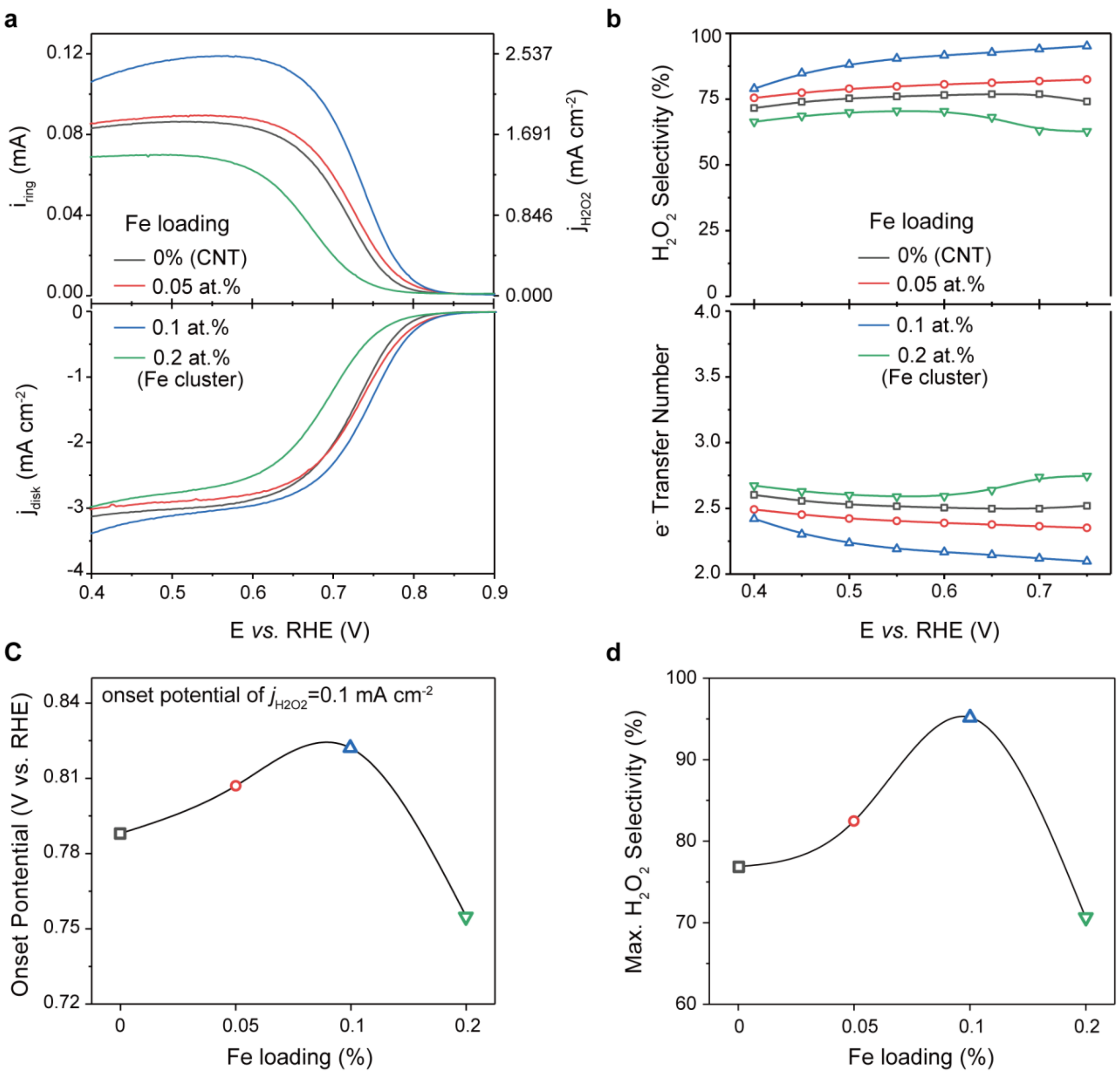

Supplementary Figure 17. Fe content effect for H_2O_2 generation. (**a**) Linear sweep voltammetry of CNT and Fe-CNT with different Fe loading, (**b**) calculated H_2O_2 selectivity and electron transfer number during potential sweep, (**c**) onset potential and (**d**) maximum H_2O_2 selectivity as a function of Fe loading. It is noted that introducing trace amount of Fe atoms boosts the ORR kinetics and improves the H_2O_2 selectivity, while the formation of iron oxide clusters in addition to Fe single atom (Supplementary Note 1, Supplementary Figs. 2-3) decreases the O_2-to-H_2O_2 conversion performance.

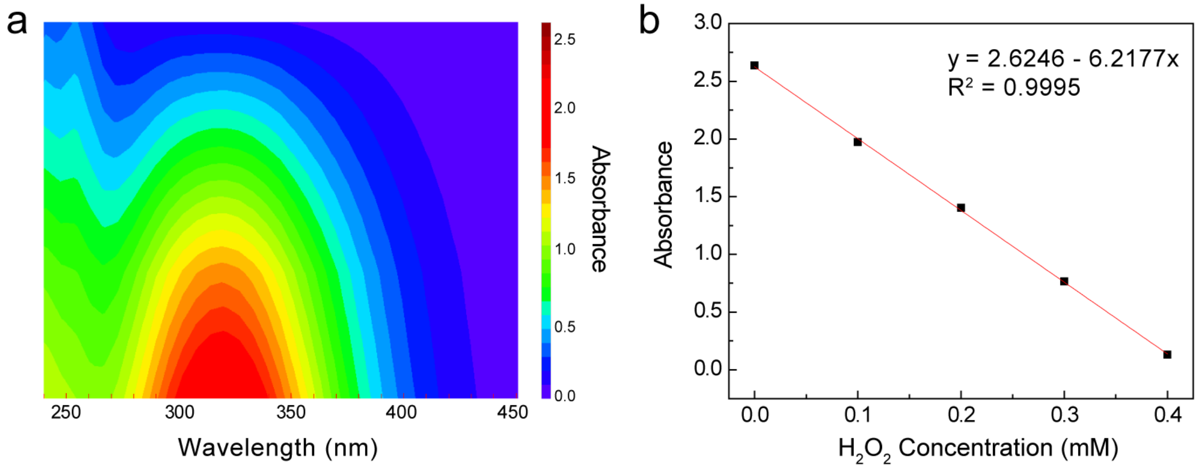

Supplementary Figure 18. Colorimetric method calibration. (**a**) Contour map of UV-Vis absorption spectra for cerium titration by known H_2O_2 concentration and (**b**) linear fitting of absorbance at 320 nm wavelength.

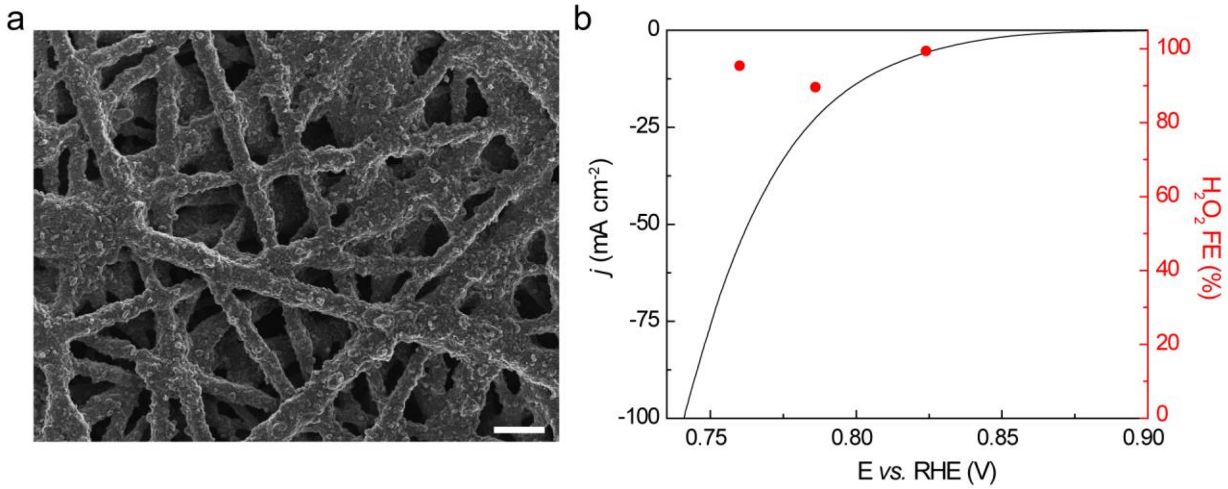

Supplementary Figure 19. Bulk electrolysis for H_2O_2 generation in a homemade H-cell electrolyzer. (**a**) SEM image of GDL supported catalyst at a loading of 0.5 mg cm^{-2}. Scale bar, 50 µm. (**b**) Polarization curve of Fe-CNT/GDL catalyst in 1 M KOH electrolyte with manual *i*R compensation, together with 3 representative H_2O_2 selectivity measurements after ~ 10-min chronoamperometric electrolysis at different potentials. Sweep rate of 5 mV s^{-1}. At 0.76 V *vs.* RHE, the steady-state overall current is ~ 45.1 mA cm^{-2}, slightly lower than the polarization value which has also been observed in many other catalytic reactions such as HER or CO_2RR. The H_2O_2 partial current was measured to be 43 mA cm^{-2} with a Faradaic efficiency of 95.4%, corresponding to a H_2O_2 generation rate of ~ 1.6 mol g^{-1} h^{-1} or 8.0 mol m^{-2} h^{-1} .

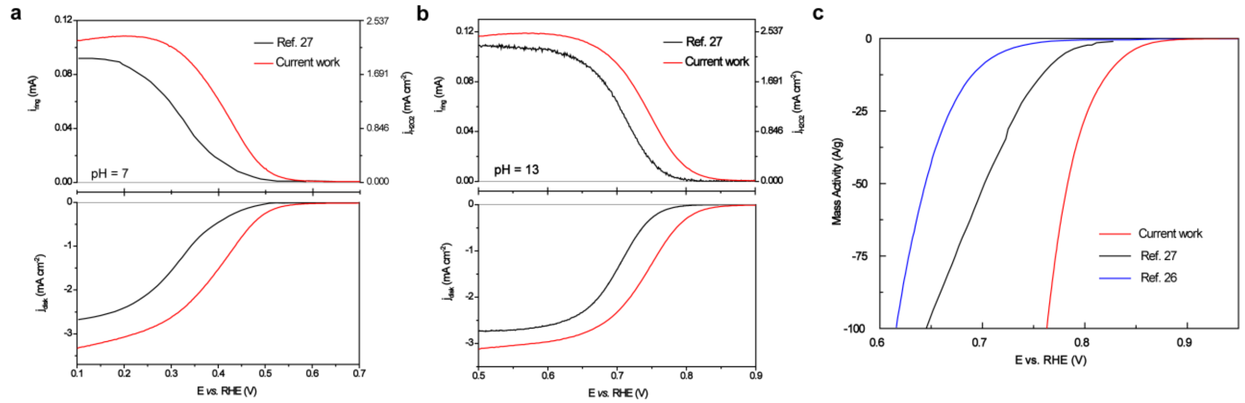

Supplementary Figure 20. O_2-to-H_2O_2 performance comparison of current work with 2 latest reports. (**a, b**) RRDE results comparison on our Fe-CNT vs. O-CNT reported in Ref. 27. (**c**) Mass-activity comparison of our Fe-CNT vs. F-mrGO_P50 of Ref. 26 and O-CNT of Ref. 27. Please note that the geometric current density was present in Supplementary Fig. 15, while in this current figure both Fe-CNT and O-CNT of Ref. 27 (a same catalyst loading of 0.5 mg/cm^2) was normalized to mass activity in comparison to Ref. 26 (a catalyst loading of 0.01 mg/cm^2). The F-mrGO_P50 catalyst in Ref. 26 was carried out in 0.1 M KOH, while the other two catalysts were tested in 1 M KOH. The possible mechanism was explained in Supplementary Fig. 25.

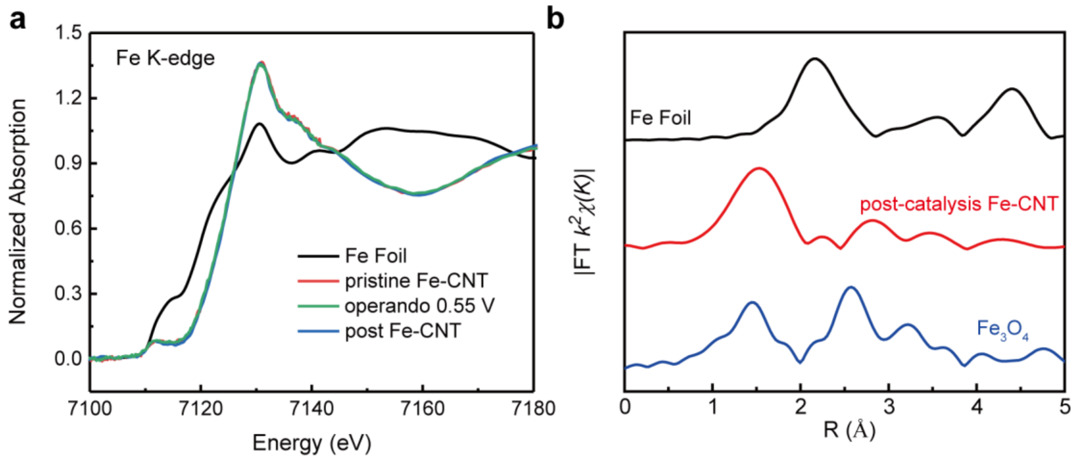

Supplementary Figure 21. Structural stability characterization by Fe K-edge XAS. (**a**) XANES comparison of before, during, and after 2-h's continuous ORR electrolysis at 0.55 V *vs.* RHE and (**b**) EXAFS of post-catalysis Fe-CNT with Fe metal and Fe_3O_4 as references.

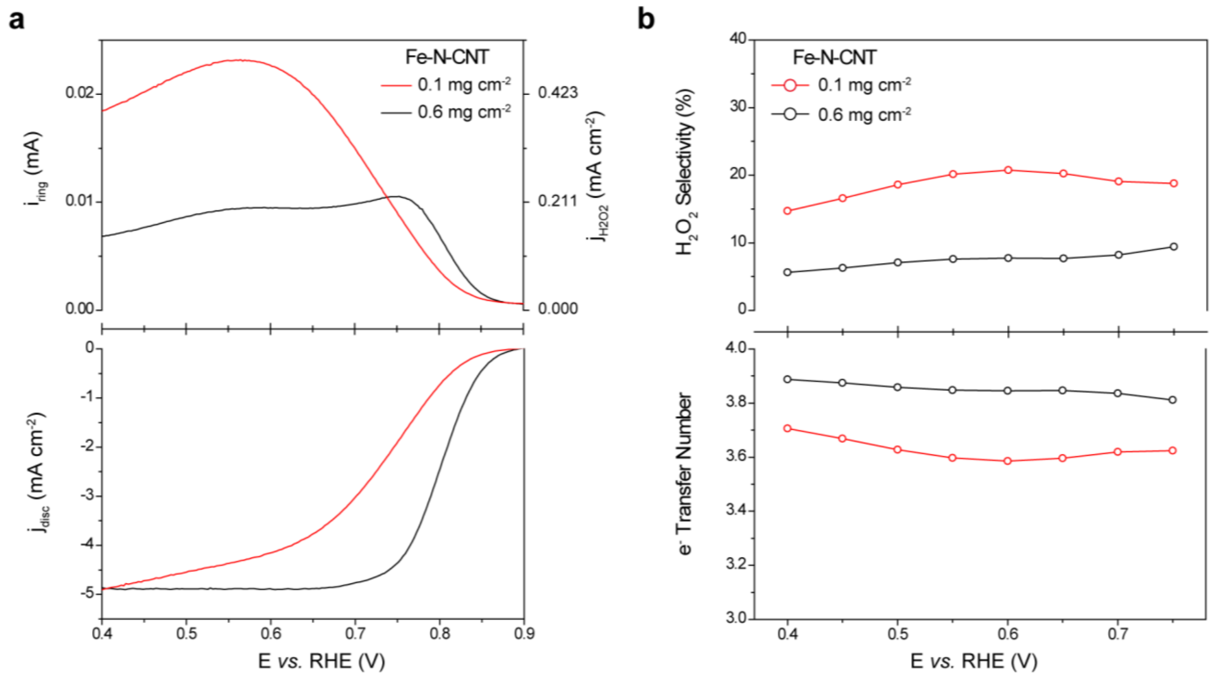

Supplementary Figure 22. Catalyst loading effect on ORR pathway. (**a**) Linear sweep voltammetry of oxygen reduction over Fe-N-CNT catalyst at different loading (red line: 0.1 mg cm^{-2}, a typical loading for H_2O_2 generation comparison; black line: 0.6 mg cm^{-2}, a typical loading for fuel cell test[13,17]), scan rate of 5 mV s^{-1}, rotating speed of 1600 rpm, ring electrode potential of 1.2 V *vs.* RHE. (**b**) Calculated H_2O_2 selectivity and electron transfer number during potential sweep. The electron transfer number increased from 3.7 to 3.9 (H_2O_2 yield ~5%) with increasing Fe-N-CNT catalyst loading, in good agreement with earlier Fe-N-C results[14,15].

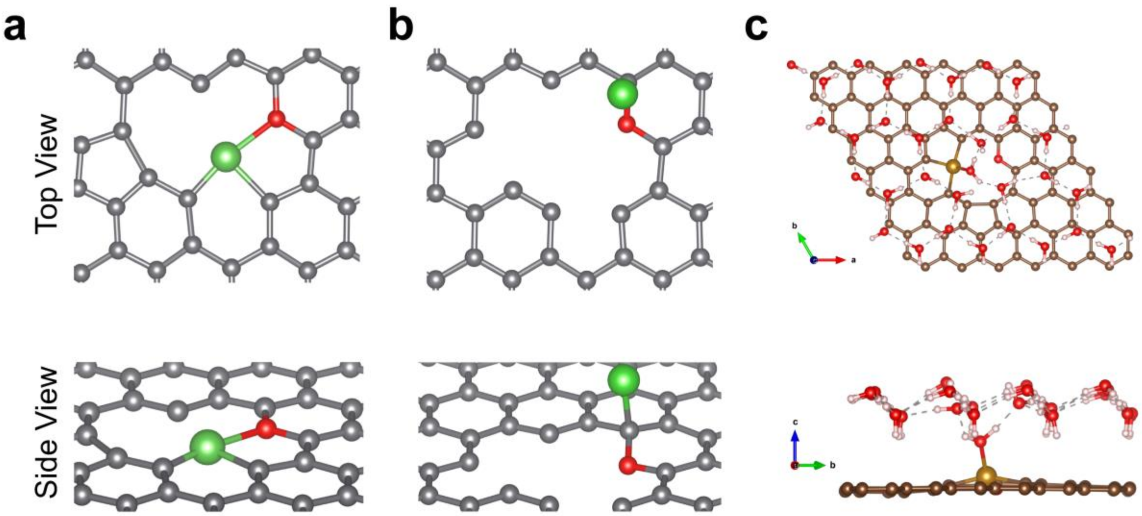

Supplementary Figure 23. Coordination structure illustration of Fe-C-O motifs. The in-plane (**a**), out-of-plane (**b**), and in the presence of a water monolayer (**c**) atomic structures of Fe-C-O 5V-O_1 motif as a representative example. Fe atoms "sucked" into the CNT vacancies showed higher stability than those adsorbed on the surface.

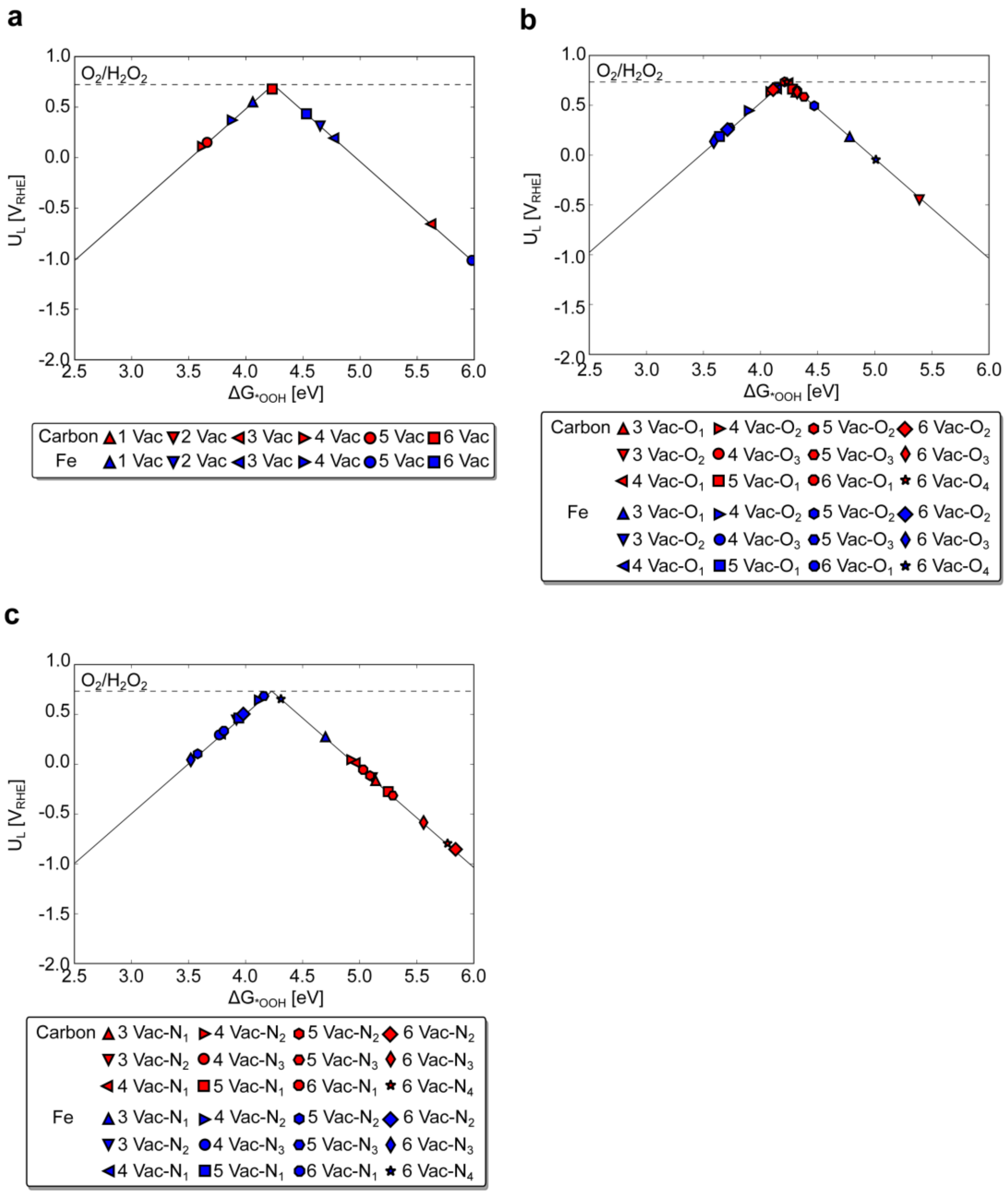

Supplementary Figure 24. Theoretical screening of various coordination motifs. Broader view of volcano plots showing the calculated 2e$^-$ ORR limiting potential (U_L) as a function of OOH* adsorption energy for (**a**) Fe-C, (**b**) Fe-C-O and (**c**) Fe-C-N systems plotted as a function of *OOH binding free energies (ΔG_{*OOH}). Red and blue symbols indicate *OOH adsorption at C and Fe atoms, respectively.

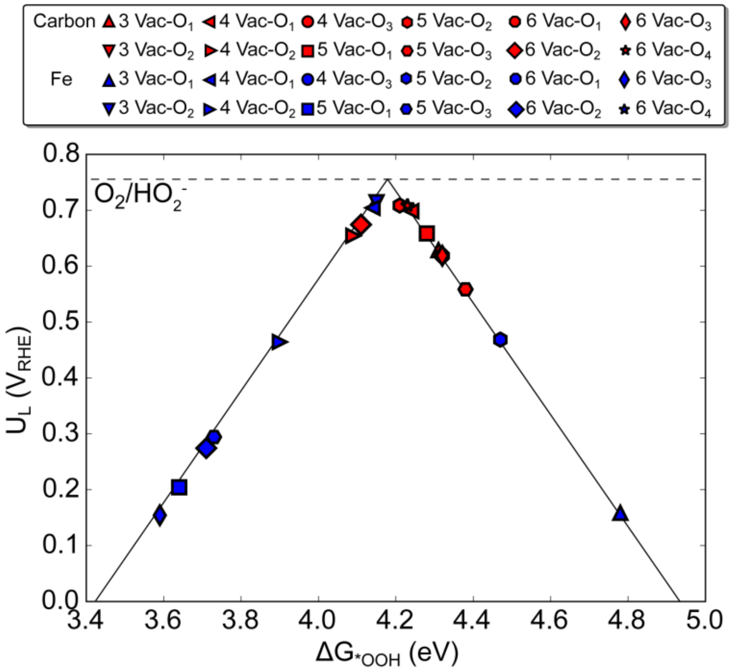

Supplementary Figure 25. The corresponding volcano plot of HO_2^- formation with a 0.75 V *vs.* RHE equilibrium potential in pH > 11.6 solutions. In the theoretical model, the difference in the equilibrium potential only results in the change of the peak position by 0.07 eV, which does not change the general trends and conclusions of our active sites identification.

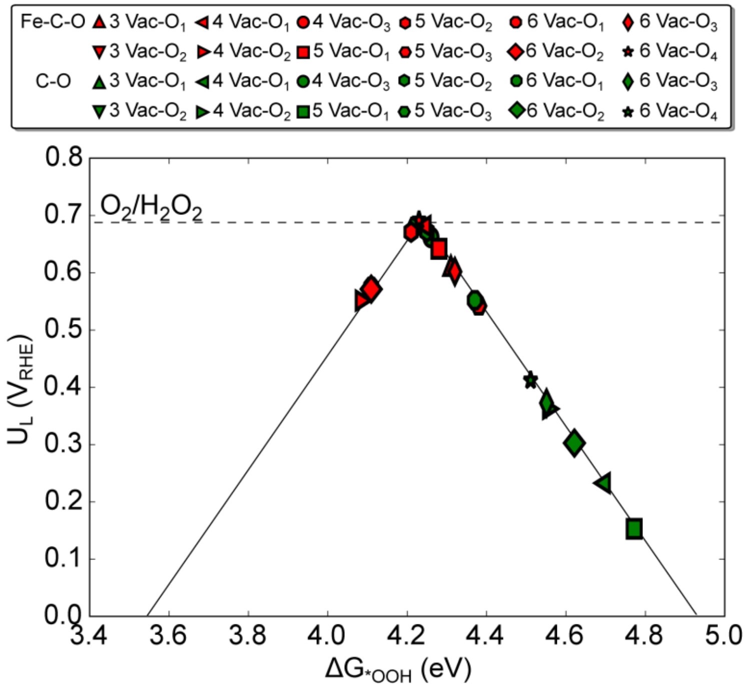

Supplementary Figure 26. Calculated ORR activity volcano plot for 2e⁻ pathway to H_2O_2 on C-O and Fe-C-O motifs. Red and green symbols indicate *OOH adsorption at carbon sites of Fe-C-O and C-O motifs, respectively. It suggests that with the incorporation of atomic Fe, the binding strength of *OOH gets strengthened toward the peak of volcano plot for more selective O_2-to-H_2O_2 conversion.

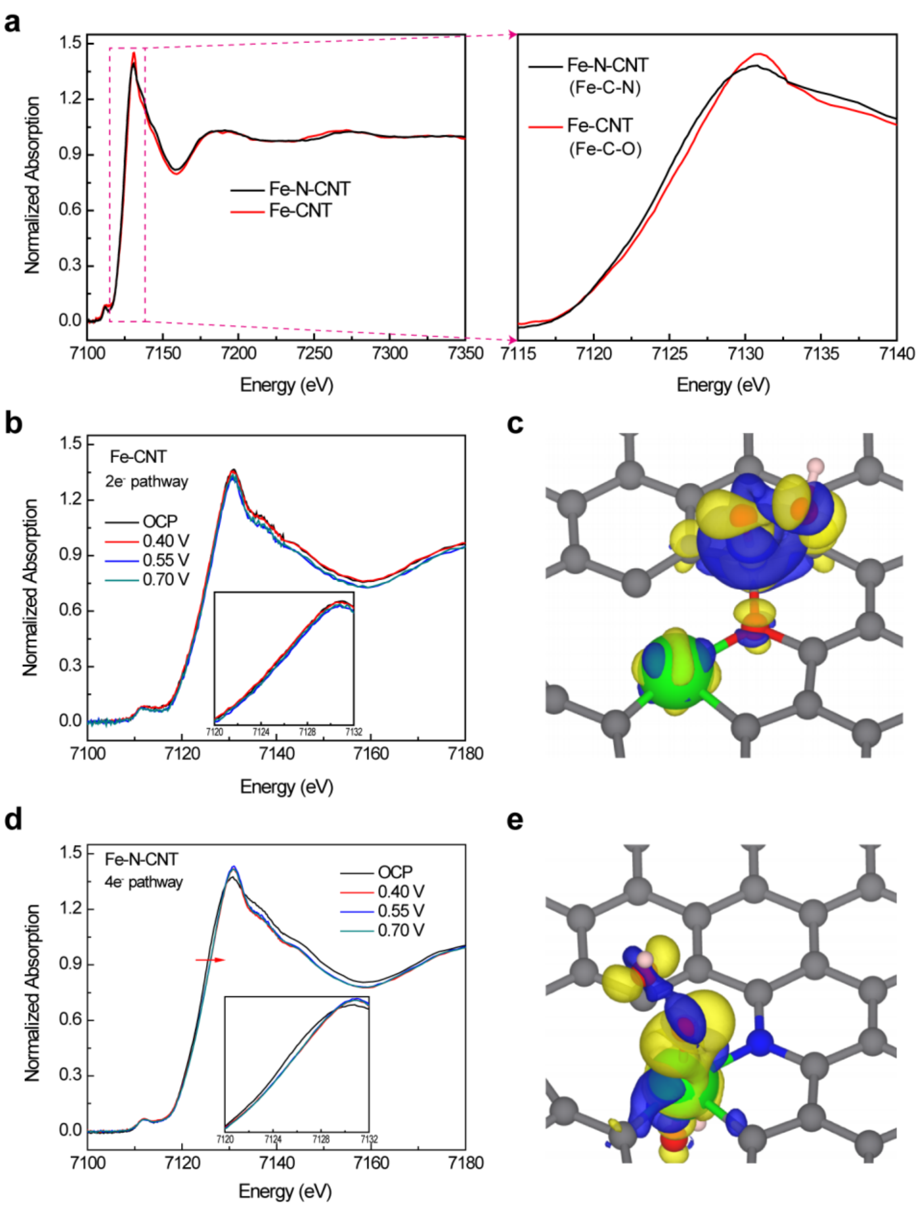

Supplementary Figure 27. Operando XAS characterization and DFT simulations on Fe-C-O vs. Fe-C-N. (**a**) Comparison between the ex-situ Fe K-edge XANES of Fe-CNT (red line) and Fe-N-CNT (black line). Fe-N-CNT shows similar feature compared to earlier reports[16-18]. A slightly higher oxidation state of Fe was observed in Fe-C-O motifs than that of Fe-C-N. (**b**) *In-Operando* Fe K-edge XANES of Fe-CNT and (**d**) Fe-N-CNT under electrochemical ORR conditions, and corresponding charge density plots upon (**c**) Fe-O-C-(*OOH) and (**e**) (HOO*)-Fe-N-C motifs. The isosurface level was set to 0.005 e/$Å^3$. It is noted that the oxidation state of Fe remained unshifted in Fe-CNT during potential sweep, suggesting no additional Fe-O bonding formed during the reaction conditions. In contrast, a salient near edge shift is observed on Fe-N-CNT, which is arisen from the more pronounced charge redistribution upon *OOH adsorption on the Fe site of Fe-N-C coordination.

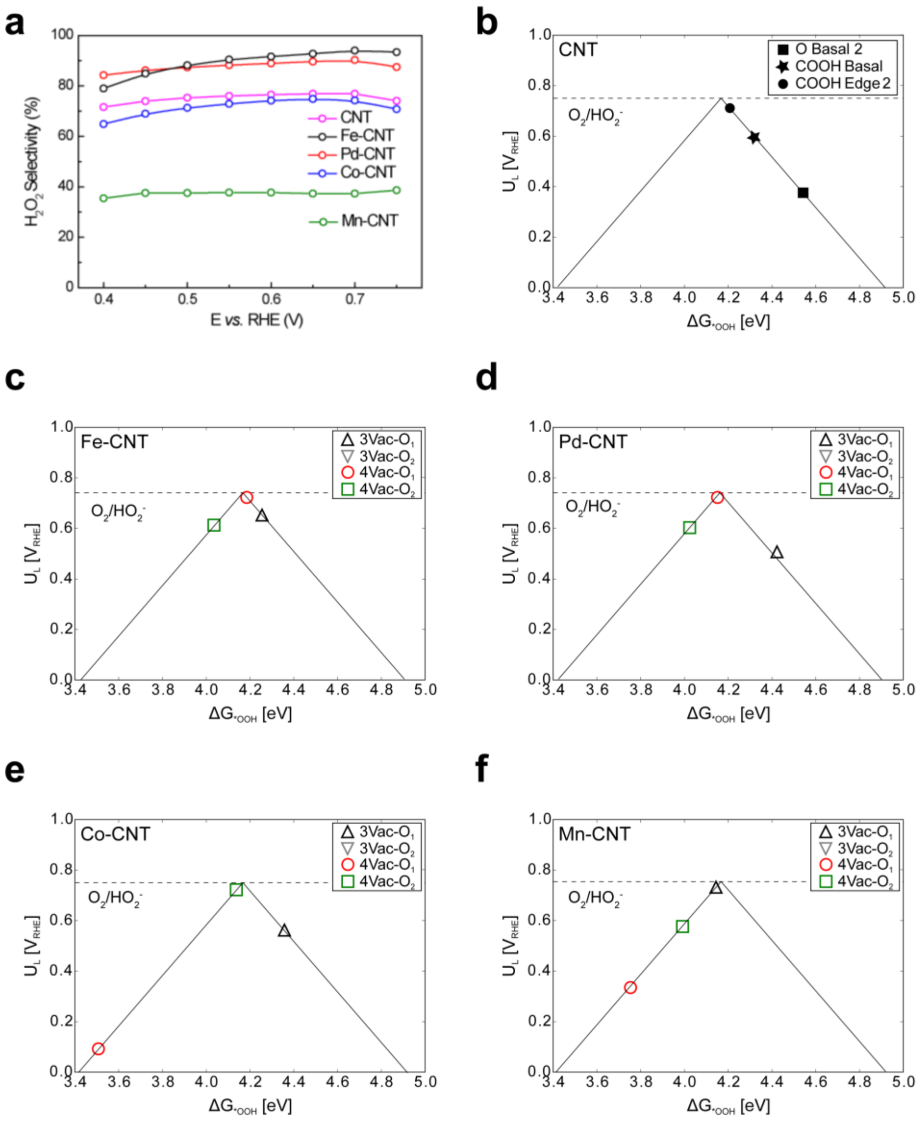

Supplementary Figure 28. Comparison of experimental and theoretical results in the trend of H_2O_2 selectivity on M-CNT catalysts as well as CNT supports. The equilibrium potential we considered is 0.75 V *vs.* RHE as the comparison was performed in strong alkaline solution. (**a**) H_2O_2 selectivity of Fe-CNT, Pd-CNT, Co-CNT, Mn-CNT, and annealed CNT support in experiments. (**b-f**) Theoretical volcano plots predicting the limiting potential (U_L) of O_2 reduction reaction to H_2O_2 plotted as a function of *OOH binding free energies (ΔG_{*OOH}) on carbon sites for four representative systems of several metal atom doped and undoped CNT. We note that 3Vac-O_2 configurations are out of range. Since the as received CNT was functionalized with -COOH groups, which could turn into O dopants after the annealing process, therefore we adapted three possible sites from Ref. 2 in here for comparison (we only considered O dopants in vacancies larger than single vacancy which have extra room for the following Fe doping). While Fe and Pd present sites near the top of the volcano, the sites in annealed CNT present weaker binding energy with *OOH, which might be responsible for the lower H_2O_2 performance. The other two samples, Co-CNT and particularly Mn-CNT, showed sites with much stronger binding with *OOH which could dissociate O-O bond into H_2O and lower the H_2O_2 selectivity.

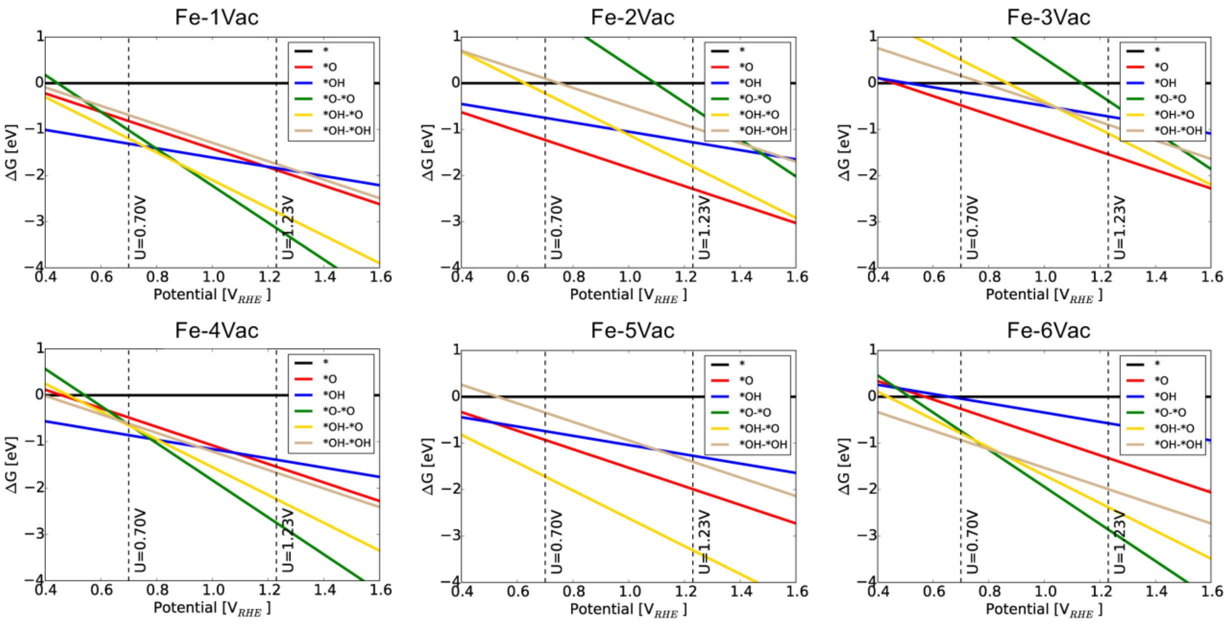

Supplementary Figure 29. **Surface Pourbaix diagram to determine the most stable coverage at 0.7 V over different Fe-C motifs.**

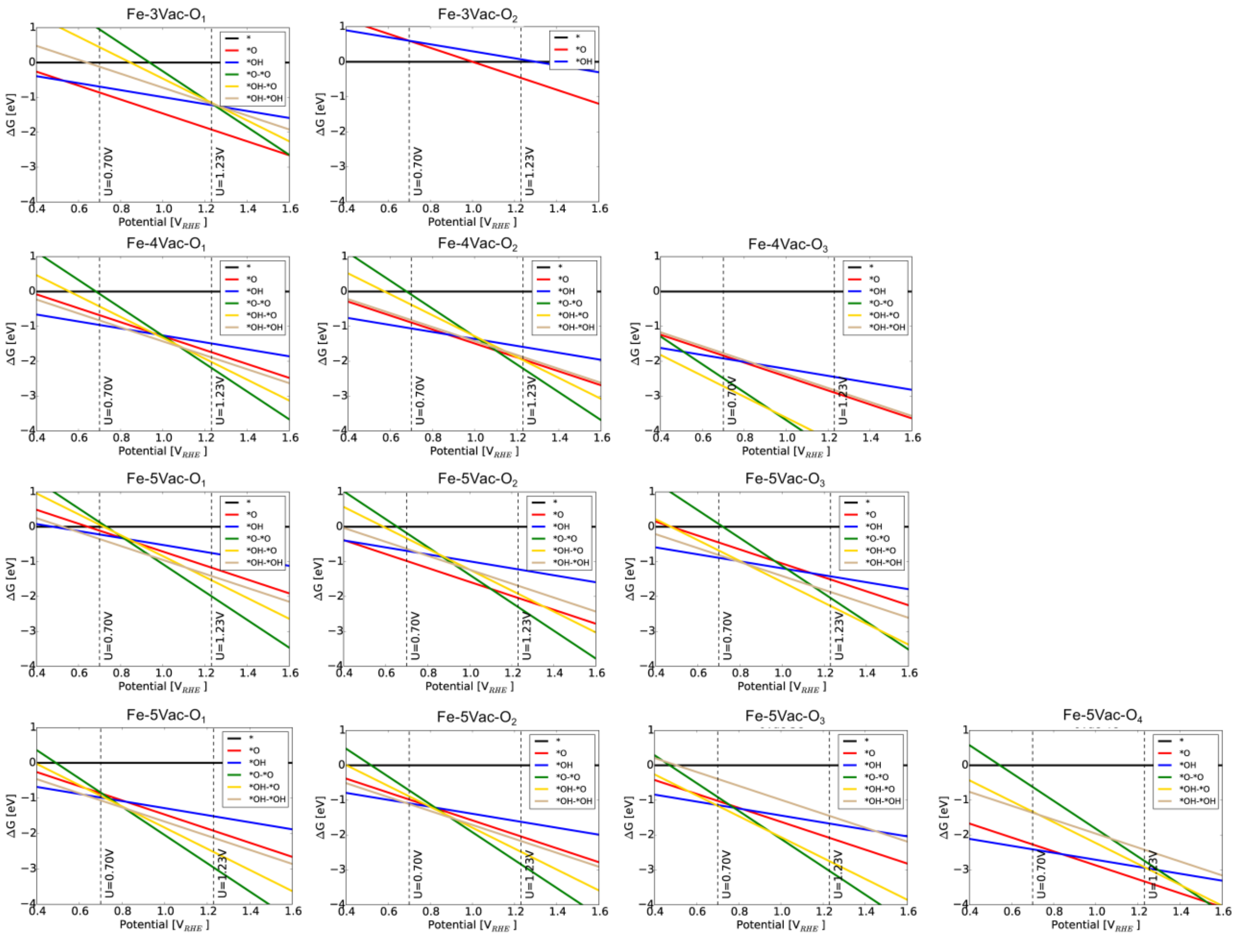

Supplementary Figure 30. Surface Pourbaix diagram to determine the most stable coverage at 0.7 V over different Fe-C-O motifs.

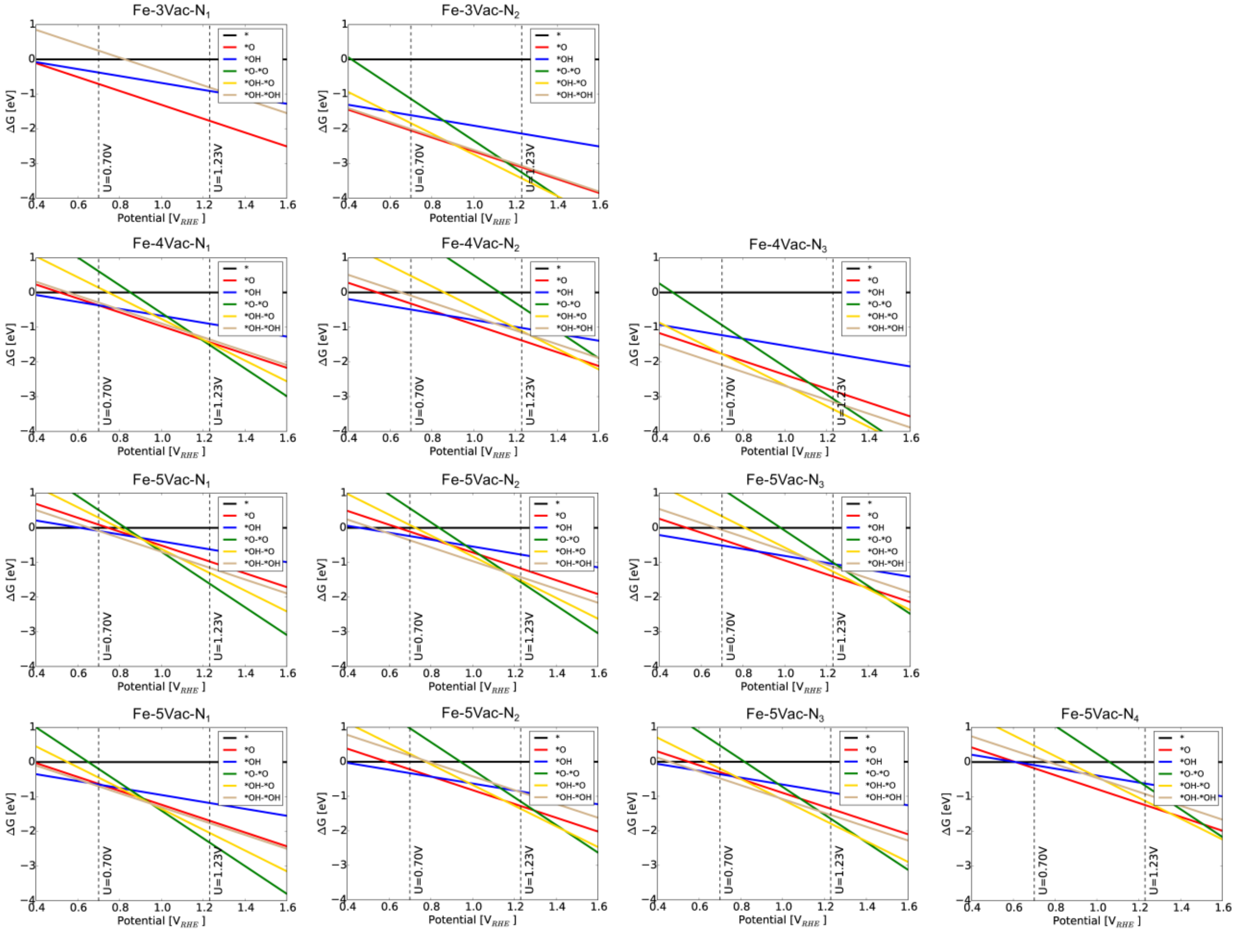

Supplementary Figure 31. Surface Pourbaix diagram to determine the most stable coverage at 0.7 V over different Fe-C-N motifs.

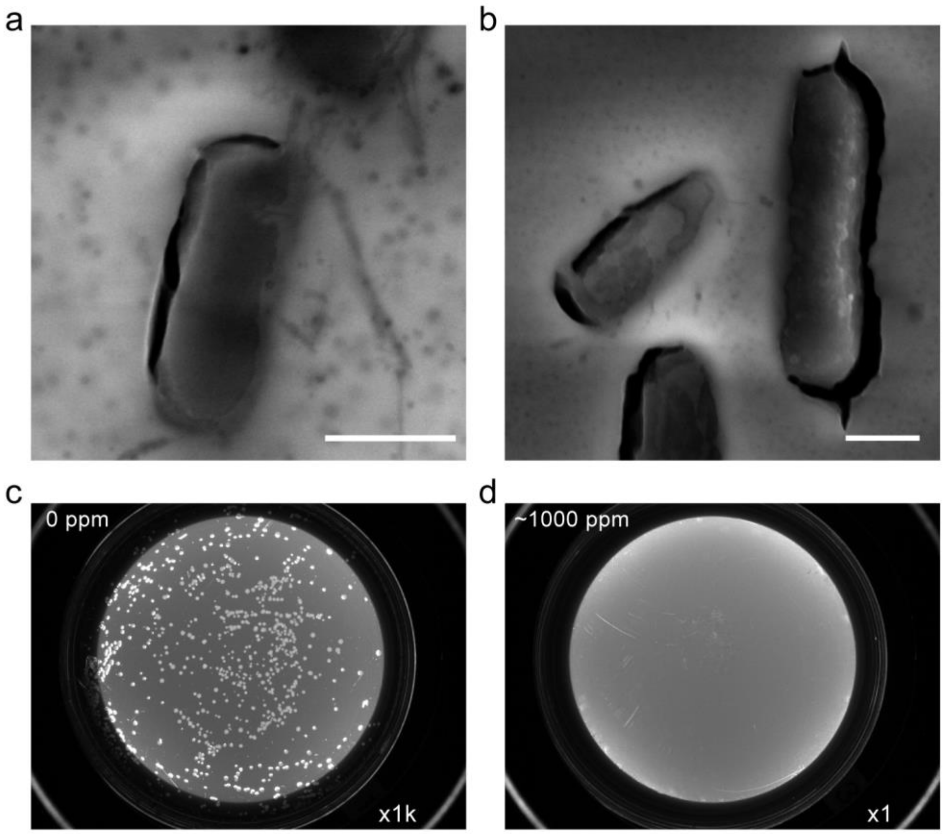

Supplementary Figure 32. *E. coli* bacterial Characterizations. (**a**, **b**) SEM images of *E. coli* bacterial (**a**) before and (**b**) after H_2O_2 electrolysis. (**c**, **d**) CCD photos of overnight cultured plates with spread droplets taken from E Coli contaminated 0.1 M PBS solution. Generated H_2O_2 concentration is labelled at the upper left corner, with dilution factor labelled at the right bottom corner of each image. The 0 ppm image is adopted from Fig. 5f. An effective water disinfection of >99.9999% bacteria removal is demonstrated by mixing electrochemical synthesized H_2O_2 solution with the bacteria without potential applied.

Supplementary Table 1. Representative Bader charges for M-C-O in 4V-O_1 coordination.

Motif	Fe-C-O	Pd-C-O	Co-C-O	Mn-C-O
Charge	0.89	0.40	0.61	1.09

Supplementary Table 2. The calculated formation energies of representative Fe single atom configurations and surface adsorption of Fe atom.

(eV)	Single atom configuration	Surface adsorption
5V-O_1	-1.66	3.47
5V-O_2	-2.02	3.62
4V-N_2	-2.09	Relaxed to SAC
5V-N_3	-2.38	Relaxed to SAC

Supplementary References

1. Do, S.-H., Batchelor, B., Lee, H.-K. & Kong, S.-H. Hydrogen peroxide decomposition on manganese oxide (pyrolusite): Kinetics, intermediates, and mechanism. *Chemosphere* **75**, 8-12 (2009).
2. Kitajima, N., Fukuzumi, S. & Ono, Y. Formation of superoxide ion during the decomposition of hydrogen peroxide on supported metal oxides. *J. Phys. Chem.* **82**, 1505-1509 (1978).
3. Back, S., Kulkarni, A.R. & Siahrostami, S. Single Metal Atoms Anchored in Two-Dimensional Materials: Bifunctional Catalysts for Fuel Cell Applications. *ChemCatChem* **10**, 3034-3039 (2018).
4. Sarapuu, A., Vaik, K., Schiffrin, D.J. & Tammeveski, K. Electrochemical reduction of oxygen on anthraquinone-modified glassy carbon electrodes in alkaline solution. *J. Electroanal. Chem.* **541**, 23-29 (2003).
5. Jirkovský, J.S. *et al.* Single Atom Hot-Spots at Au–Pd Nanoalloys for Electrocatalytic H2O2 Production. *J. Am. Chem. Soc.* **133**, 19432-19441 (2011).
6. Lee, Y.-H., Li, F., Chang, K.-H., Hu, C.-C. & Ohsaka, T. Novel synthesis of N-doped porous carbons from collagen for electrocatalytic production of H2O2. *Appl. Catal., B* **126**, 208-214 (2012).
7. Siahrostami, S. *et al.* Enabling direct H2O2 production through rational electrocatalyst design. *Nat. Mater.* **12**, 1137-1143 (2013).
8. Verdaguer-Casadevall, A. *et al.* Trends in the Electrochemical Synthesis of H2O2: Enhancing Activity and Selectivity by Electrocatalytic Site Engineering. *Nano Lett.* **14**, 1603-1608 (2014).
9. Choi, C.H. *et al.* Tuning selectivity of electrochemical reactions by atomically dispersed platinum catalyst. *Nat. Commun.* **7**, 10922 (2016).
10. Zhaoke, Z., Hau, N.Y., Da-Wei, W. & Rose, A. Epitaxial Growth of Au–Pt–Ni Nanorods for Direct High Selectivity H2O2 Production. *Adv. Mater.* **28**, 9949-9955 (2016).
11. Lu, Z. *et al.* High-efficiency oxygen reduction to hydrogen peroxide catalysed by oxidized carbon materials. *Nat. Catal.* **1**, 156-162 (2018).
12. Sun, Y. *et al.* Efficient Electrochemical Hydrogen Peroxide Production from Molecular Oxygen on Nitrogen-Doped Mesoporous Carbon Catalysts. *ACS Catal.* **8**, 2844-2856 (2018).
13. Lefèvre, M., Proietti, E., Jaouen, F. & Dodelet, J.-P. Iron-Based Catalysts with Improved Oxygen Reduction Activity in Polymer Electrolyte Fuel Cells. *Science* **324**, 71-74 (2009).
14. Bonakdarpour, A. *et al.* Impact of Loading in RRDE Experiments on Fe–N–C Catalysts: Two- or Four-Electron Oxygen Reduction? *Electrochem. Solid-State Lett.* **11**, B105-B108 (2008).
15. Wu, G., More, K.L., Johnston, C.M. & Zelenay, P. High-Performance Electrocatalysts for Oxygen Reduction Derived from Polyaniline, Iron, and Cobalt. *Science* **332**, 443-447 (2011).
16. Zitolo, A. *et al.* Identification of catalytic sites for oxygen reduction in iron- and nitrogen-doped graphene materials. *Nat. Mater.* **14**, 937 (2015).
17. Jia, Q. *et al.* Experimental Observation of Redox-Induced Fe–N Switching Behavior as a Determinant Role for Oxygen Reduction Activity. *ACS Nano* **9**, 12496-12505 (2015).
18. Kramm, U.I. *et al.* On an Easy Way To Prepare Metal–Nitrogen Doped Carbon with Exclusive Presence of MeN4-type Sites Active for the ORR. *J. Am. Chem. Soc.* **138**, 635-640 (2016).
