# Supplementary material for: Highly selective oxygen reduction to hydrogen peroxide on transition metal single atom coordination
Source: Nat Commun. 2019 Sep 5;10:3997. doi: 10.1038/s41467-019-11992-2 (PMC6728328; doi:10.1038/s41467-019-11992-2)
Supplement: Supplementary file 3 — Description of Additional Supplementary Files [file 41467_2019_11992_MOESM3_ESM.pdf]

Description of Additional Supplementary Files

File Name: Supplementary Movie 1

Description: Reconstructed tomography of the Fe-CNT sample tip. Atom labels, Au in yellow, Fe in green, C in grey, and O in red.
