# Supplementary material for: Highly selective oxygen reduction to hydrogen peroxide on transition metal single atom coordination
Source: Nat Commun. 2019 Sep 5;10:3997. doi: 10.1038/s41467-019-11992-2 (PMC6728328; doi:10.1038/s41467-019-11992-2)
Supplement: Supplementary file 2 — Peer Review File [file 41467_2019_11992_MOESM2_ESM.pdf]

Editorial Note: This manuscript has been previously reviewed at another journal that is not operating a transparent peer review scheme. This document only contains reviewer comments and rebuttal letters for versions considered at *Nature Communications*.

REVIEWERS' COMMENTS:

Reviewer #1 (Remarks to the Author):

The authors addressed my concerns, and I recommend to publish the manuscript as is.

Response to reviewers' comments:

Reviewer #1

"The authors addressed my concerns, and I recommend to publish the manuscript as is."

--- We thank the reviewer's positive response.
